# Interleukin-like epithelial-to-mesenchymal transition inducer activity is controlled by proteolytic processing and plasminogen–urokinase plasminogen activator receptor system–regulated secretion during breast cancer progression

**DOI:** 10.1186/s13058-014-0433-7

**Published:** 2014-09-09

**Authors:** Agnes Csiszar, Betül Kutay, Silvia Wirth, Ulrike Schmidt, Sabine Macho-Maschler, Martin Schreiber, Memetcan Alacakaptan, Georg F Vogel, Karin Aumayr, Lukas A Huber, Hartmut Beug

**Affiliations:** 1 Research Institute of Molecular Pathology, Dr. Bohr-Gasse 7, Vienna, A-1030 Austria; 2 Department of Biomedical Sciences, Institute of Animal Breeding and Genetics, University of Veterinary Medicine, Veterinaerplatz 1, Vienna, A-1210 Austria; 3 Department of Obstetrics and Gynecology, Medical University of Vienna, Wäringergürtel 18-20, Vienna, A-1090 Austria; 4 Division of Histology and Embryology, Innsbruck Medical University, Müllerstrasse 59, Innsbruck, A-6020 Austria; 5 Biocenter, Division of Cell Biology, Innsbruck Medical University, Innrain 80-82, Innsbruck, A-6020 Austria; 6 Swiss Institute for Experimental Cancer Research (ISREC), School of life Sciences, Swiss Federal Institute of Technology of Lausanne (EPFL), Station 19, Lausanne, 1015 Switzerland; 7 Institute of Cancer Research, Department of Medicine I, Medical University of Vienna, Borschkegasse 8a, Vienna, A-1090 Austria

## Abstract

**Introduction:**

Interleukin-like epithelial-to-mesenchymal transition inducer (ILEI) is an essential cytokine in tumor progression that is upregulated in several cancers, and its altered subcellular localization is a predictor of poor survival in human breast cancer. However, the regulation of ILEI activity and the molecular meaning of its altered localization remain elusive.

**Methods:**

The influence of serum withdrawal, broad-specificity protease inhibitors, different serine proteases and plasminogen depletion on the size and amount of the secreted ILEI protein was investigated by Western blot analysis of EpRas cells. Proteases with ILEI-processing capacity were identified by carrying out an *in vitro* cleavage assay. Murine mammary tumor and metastasis models of EpC40 and 4T1 cells overexpressing different mutant forms of ILEI were used—extended with *in vivo* aprotinin treatment for the inhibition of ILEI-processing proteases—to test the *in vivo* relevance of proteolytic cleavage. Stable knockdown of urokinase plasminogen activator receptor (uPAR) in EpRas cells was performed to investigate the involvement of uPAR in ILEI secretion. The subcellular localization of the ILEI protein in tumor cell lines was analyzed by immunofluorescence. Immunohistochemistry for ILEI localization and uPAR expression was performed on two human breast cancer arrays, and ILEI and uPAR scores were correlated with the metastasis-free survival of patients.

**Results:**

We demonstrate that secreted ILEI requires site-specific proteolytic maturation into its short form for its tumor-promoting function, which is executed by serine proteases, most efficiently by plasmin. Noncleaved ILEI is tethered to fibronectin-containing fibers of the extracellular matrix through a propeptide-dependent interaction. In addition to ILEI processing, plasmin rapidly increases ILEI secretion by mobilizing its intracellular protein pool in a uPAR-dependent manner. Elevated ILEI secretion correlates with an altered subcellular localization of the protein, most likely representing a shift into secretory vesicles. Moreover, altered subcellular ILEI localization strongly correlates with high tumor cell–associated uPAR protein expression, as well as with poor survival, in human breast cancer.

**Conclusions:**

Our findings point out extracellular serine proteases, in particular plasmin, and uPAR as valuable therapeutic targets against ILEI-driven tumor progression and emphasize the prognostic relevance of ILEI localization and a combined ILEI-uPAR marker analysis in human breast cancer.

**Electronic supplementary material:**

The online version of this article (doi:10.1186/s13058-014-0433-7) contains supplementary material, which is available to authorized users.

## Introduction

Metastasis of carcinomas is responsible for most cancer-associated deaths [1], yet it is still the least understood part of cancer pathogenesis. This illustrates the need for a better understanding of the molecular mechanisms driving tumor progression. The first permissive step of metastasis is the physical dissemination of cancer cells from the primary tumor, which is orchestrated by the process of epithelial-to-mesenchymal transition (EMT) [2]. Tumor cells in advanced primary carcinomas recruit a variety of cell types into the surrounding stroma, which, in response, release many EMT-inducing [1] and other tumor-promoting signals [3],[4]. Autocrine signaling loops are later established by cancer cells to maintain EMT on the voyage through different tissue environments [5],[6].

Interleukin-like epithelial-to-mesenchymal transition inducer (ILEI), also called FAM3C, is one of the secreted factors that regulate tumor progression [7]. ILEI was found in an expression profiling screen for transforming growth factor β (TGFβ)-regulated EMT-specific genes [8]; it is upregulated by TGFβ at the translational level [7],[9],[10]. Stable overexpression of ILEI in several murine tumor cell lines induces EMT *in vitro* and elevated tumor growth and metastasis *in vivo*[7],[11]. Furthermore, RNA interference (RNAi)-mediated knockdown (KD) of the protein prevents or reverses TGFβ-dependent EMT and metastasis formation [7],[10]. Recombinant ILEI induces EMT *in vitro*, suggesting that it is the secreted protein responsible for ILEI function [7]. However, the failure to produce bioactive recombinant ILEI in large quantities indicates that crucial characteristics of its active form are still unknown. Contrary to the originally described interleukin (IL)-like fold [12], only a recent structural study shed light onto ILEI/FAM3C and its family representing a new structural class of noncytokine folds [13]. Importantly, ILEI is overexpressed in several human tumors and shows altered subcellular localization (granular to cytoplasmic), which strongly correlates with metastasis formation and survival in human breast and hepatocellular carcinomas [7],[11]. However, the molecular distinction between granular and cytoplasmic ILEI is still not understood.

Proteases have an important role during malignant progression by acting as an extensive multidirectional network of proteolytic interactions [14]. The plasminogen (Plg)–Plg activator (PA) system is essential in extracellular matrix (ECM) degradation, in the release and activation of several growth factors, such as TGFβ1 [15] and platelet-derived growth factors [16]-[18], as well as in a number of cellular responses through the activation of urokinase plasminogen activator receptor (uPAR) signaling [19],[20]. Plg can be converted into plasmin by the serine proteases urokinase plasminogen activator (uPA) and tissue plasminogen activator (tPA) [14]. Activation of uPA is dependent on the binding of pro-uPA to its specific membrane receptor uPAR and can be achieved by plasmin itself, thus creating a feedback loop of reciprocal activation [19]. Alternatively, kallikrein can activate both Plg [21] and uPA [22]. Important negative regulators of the cascade are plasminogen activator inhibitor 1 (PAI-1) and PAI-2 [23]. The cellular responses are mediated by direct contact of uPAR with a variety of extracellular proteins and membrane receptors, which results in the activation of diverse intracellular signaling molecules [24]. Plasma kallikrein also activates inflammation as part of the kallikrein–kinin system [25]. Proteases of recruited inflammatory cells, such as neutrophil elastase (NE), propagate angiogenesis and invasiveness by ECM degradation and induction of tumor cell proliferation [26]. In agreement with their role in cancer progression and metastasis, increased expression of uPA, uPAR, PAI-1 and NE, and a positive correlation between their levels and a poor prognosis, have frequently been reported in malignant tumors [26],[27].

In this study, we show how proteases, in particular the Plg-uPAR system, are involved at multiple levels in the regulation of ILEI activity and the consequences of these mechanisms on primary tumor growth and metastasis-free survival, in both mouse and human models.

## Methods

### Cell culture

The EpRas and EpC40 cell lines, which are derivatives of the immortalized murine mammary epithelial cell line EpH4, were generated and characterized earlier in our laboratory. EpRas is tumorigenic and metastatic because of its transformation with oncogenic Ras (RasV12) [28], whereas EpC40 is tumorigenic but not metastatic because of the transformation with an effector-loop mutant Ras oncogene (RasV12C40), which activates only the phosphatidylinositol 3-kinase pathway [29],[30]. EpRas and EpC40 cells and their ILEI-overexpressing derivatives were cultivated and recultivated from mouse tumors as described previously [29],[31]. Upon serum withdrawal, 10% fetal calf serum (FCS) was replaced by 0.1% bovine serum albumin. 4T1 cells were provided by Robert Weinberg and were cultivated, including their RNAi-KD and ILEI-overexpressing derivatives, as he and his colleagues previously described [31]. Human breast cancer cell lines MCF7, T47D, MDA-MB-468, MDA-MB-231 and CAMA1 were donated by Gerhard Wirl and were cultivated as he and his research group described elsewhere [32]. NIH3T3 cells were provided by Boehringer Ingelheim and were cultured in Dulbecco’s modified Eagle’s medium (DMEM) supplemented with 10% FCS.

### Generation of stable cell lines expressing different ILEI constructs and ILEI and uPAR shRNA vectors

Mouse ILEI coding sequence with a C-terminal 6xHis affinity tag or FLAG epitope tag was PCR-cloned into pcDNA3.1 using EcoRI restriction sites. ILEI constructs containing point mutations and internal deletions were generated using the *in vitro* Stratagene QuikChange Site-Directed Mutagenesis kit (Agilent Technologies, Santa Clara, CA, USA) with pcDNA3.1-ILEI-6xHis and pcDNA3.1-ILEI-FLAG as templates. pcDNA3.1-ILEI-6xHis constructs were recloned into the pMSCV-IRES-GFP vector using EcoRI restriction sites, followed by the generation of stably transfected GP + E-86 retroviral producer cell lines. Conditioned medium (CM) of the producers was used to generate stable, retrovirally infected EpRas cell lines overexpressing different ILEI-6xHis constructs. The lentiviral expression vector pWPI (plasmid 12254; Addgene, Cambridge, MA, USA) was used to generate ILEI-FLAG constructs for stable mammalian gene expression. pWPI was modified by introducing a linker into the PacI site of the vector, thereby extending the multiple cloning site (MCS) with the unique BspEI, BamHI, SpeI and SmaI restriction sites ( = pWPI-MCS). All ILEI-FLAG constructs were recloned into the lentiviral expression vector pWPI-MCS using the BamHI and SmaI restriction sites. Lentiviral transducing particles were produced by transient cotransfection of the pWPI-ILEI-FLAG constructs together with the packaging and envelope vectors psPAX2 and pMD2.G (plasmids 12260 and 12259; Addgene) [33] into 293FT cells. Forty-eight hours after transfection, CM containing the virus particles was taken to infect EpC40 and EpRas cells and RNAi-KD derivatives of the 4T1 cell line.

Sequence fidelity and the presence of the desired mutations were confirmed by DNA sequence analysis. All infected cells were sorted twice by fluorescence-activated cell sorting for stable green fluorescent protein expression. ILEI expression levels were analyzed on Western blots using anti-ILEI, anti-penta-His and anti-FLAG antibodies.

For KD of ILEI in 4T1 and uPAR in EpRas cells, MISSION short-hairpin RNA (shRNA) lentiviral transduction particles (Sigma-Aldrich, St Louis, MO, USA) were used according to the manufacturer’s instructions. Transduced cells were selected for puromycin resistance. ILEI expression was studied by Western blot analysis and uPAR expression was assessed by qRT-PCR using glyceraldehyde 3-phosphate dehydrogenase (GAPDH) expression for normalization as well as primers from QIAGEN (Hilden, Germany).

### Protease inhibitor studies on ILEI cleavage

EpRas cells were treated for 24 hours with the following inhibitors: 100 μM E64, 100 μM CA-074, 10 μM pepstatin A, 100 μM GM6001, 500 μM (4-(2-aminoethyl)-benzolsulfonylfluorid-hydrochloride) (AEBSF; Merck, Darmstadt, Germany) and 10 μM aprotinin (Lactan, Graz, Austria).

### Protein purification and immunoprecipitation

Different forms of ILEI-6xHis or ILEI-FLAG were affinity-purified using nickel-nitrilotriacetic acid (Ni-NTA) superflow resins (QIAGEN) or were immunoprecipitated using anti-FLAG M2 agarose (Sigma-Aldrich) from whole-cell lysates and CM of overexpressing EpRas cells. 6xHis affinity purification was performed under native conditions according to the manufacturer’s instructions using 5 mM, 20 mM and 150 mM imidazole (pH 8.0) in the capturing, washing and elution buffers, respectively. For FLAG immunoprecipitation, cells were lysed in phospholipase C (PLC) buffer (10% (vol/vol) glycerol, 50 mM 2-[4-(2-hydroxyethyl)piperazin-1-yl]ethanesulfonic acid (pH 7.5), 150 mM NaCl, 0.5% (vol/vol) Triton X-100, 1.5 mM MgCl_2_, 1 mM ethylene glycol tetraacetic acid, 200 μM Na_3_VO_4_ and 10 mM NaF supplemented with cOmplete Protease Inhibitor Cocktail tablets (Roche Diagnostics, Basel, Switzerland), and lysates or CM of these cells were incubated overnight with FLAG beads. Beads were washed in PLC buffer and directly boiled in 2× SDS-PAGE loading buffer for analytical coimmunoprecipitation studies or washed an additional three times in detergent-free PLC buffer, once in 150 mM NaCl, and then eluted with 0.1 M glycine (pH 2.0). Eluates were neutralized with 1.5 M Tris-HCl (pH 9.2) and used for mass spectroscopy or *in vitro* assays. During purification of ILEI proteins, which were used as a substrate for *in vitro* cleavage assays, all washing and elution steps were performed in the absence of cOmplete Protease Inhibitor Cocktail.

### In vitro ILEI cleavage assay

Purified, full-length ILEI-FLAG or ILEI-6xHis proteins were incubated with proteases (250 U of human uPA, 50 U of human tPA, 0.5 to 2.5 U of bovine thrombin, 2.5 to 12.5 mU of human plasma kallikrein and 50 μU to 5 mU of NE (Merck) and 25 μU to 5 mU of bovine plasmin (Roche Diagnostics)) at 37°C for 5 hours, followed by Western blot analysis. Equal amounts of purified, full-length ILEI-6xHis were incubated with 1-ml of serum-free medium or medium containing 10% FCS. ILEI protein was repurified from the 1-ml solutions under equal conditions via its 6xHis affinity tag using Ni-NTA spin columns (QIAGEN) according to the manufacturer’s instructions, and equal loads of eluates were analyzed on Western blots using ILEI antibody.

### ILEI secretion assay

Serum-free medium was added after cell adherence with or without the proteases listed above. For the time-course analysis, 10 ng/ml TGFβ-1 (PeproTech, Rocky Hill, NJ, USA) and 5 mU of plasmin were added to the cells directly at 12, 16, 20, 22, 23 and 23.5 hours after serum withdrawal. Aprotinin treatment (10 μM) of wild-type EpC40 (EpC40-wt) was performed for 32 hours after serum withdrawal. In all cases, CM and cells were harvested 24 hours after the last media change, and ILEI levels were determined by Western blot analysis.

### Western blot analysis

CM were concentrated via ultrafiltration using mini spin filter columns MWCO 10 kDa (Merck). Cells were harvested, and total cell numbers were determined. Gel loading of CM was normalized to the concentrated volume and total cell numbers. Loading of cell lysates was normalized to the total protein content (Bradford assay; Bio-Rad Laboratories, Hercules, CA, USA). Western blot analysis was performed using anti-ILEI [7], anti-FLAG M2 and anti-β actin antibodies (Sigma-Aldrich). Quantification of Western blots was performed using ImageJ software (National Institutes of Health, Bethesda, MD, USA).

### Plasminogen depletion of fetal calf serum

Lysine Sepharose 4B affinity medium (GE Healthcare, Chalfont St Giles, UK) was prepared, packed into columns and equilibrated with phosphate-buffered saline (pH 7.2) according to the manufacturer’s instructions. FCS was applied to the column, and flow-through was reapplied onto fresh columns an additional two times. Final flow-through was tested for residual plasmin activity using the synthetic chromogenic plasmin substrate D-Val-Leu-Lys-pNA (S-2251™; Chromogenix, Lexington, MA, USA).

### ILEI cleavage assay with blood plasma of plasminogen-knockout mice

Blood plasma of C57BL/6 *Plg*-KO mice (The Jackson Laboratory, Bar Harbor, ME, USA) and of their heterozygous and WT littermates was prepared by collecting peripheral blood into MiniCollect tubes (K3EDTA; Greiner Bio-One, Kremsmünster, Austria) and collecting the cell-free supernatant after centrifugation. Purified, WT, full-length ILEI-FLAG was incubated with 10% plasma of mice of the above-mentioned genotypes at 37°C for 5 hours, followed by ILEI Western blot analysis.

### ILEI binding assay to fibroblast-deposited extracellular matrix

Cell-free ECM deposited by NIH 3T3 fibroblasts was prepared as described elsewhere [34], incubated with different purified ILEI proteins and fixed after rigorous washing. Immunofluorescent staining was performed using fibronectin (Sigma-Aldrich) and penta-His (QIAGEN) antibodies.

### Determination of relative gene expression levels in tumor cell lines

Total RNA of MCF7, T47D, MDA-MB-468, MDA-MB-231 and CAMA1, as well as EpRas cells and its derivatives, was isolated using TRIzol reagent (Invitrogen, Carlsbad, CA, USA), and cDNA was synthesized using SuperScript II Reverse Transcriptase (Invitrogen) according to the manufacturer’s instructions. Quantitative RT-PCR was performed using the SYBR Green PCR Master Mix (Applied Biosystems, Foster City, CA, USA) and primers from QIAGEN and from the qPrimerDepot [35]. Expression levels of uPAR, uPA, tPA, PAI-1 and PAI-2 were normalized to GAPDH expression.

### ILEI colocalization studies

EpC40-wt cells with or without plasmin and aprotinin treatments, EpC40-ΔN-RS and EpC40-FD cells were fixed and immunostained with FLAG M2 (Sigma-Aldrich), GM130 (BD Biosciences, Franklin Lakes, NJ, USA), ILEI [7], early endosome antigen 1 (EEA1; BD Biosciences), giantin (Abcam, Cambridge, UK), lysosomal-associated membrane protein 1 (Lamp1, 1D4B; Developmental Studies Hybridoma Bank, Iowa City, IA, USA), TGN38 (Novus Biologicals, Littleton, CO, USA) and primary and Alexa Fluor 568 and Alexa Fluor 647 fluorochrome-coupled secondary antibodies (Invitrogen). Hoechst 33258 (Invitrogen) was used for DNA counterstaining.

For transferrin uptake studies, cells were serum-starved for 4 to 6 hours, incubated with growth medium supplemented with transferrin-Alexa Fluor 568 (Invitrogen) for 45 minutes and fixed, followed by ILEI immunostaining. For transient expression of mCherry-Rab8a protein, cells were transfected with pCherry-C1-Rab8a plasmid using Lipofectamine LTX reagent (Invitrogen) according to the manufacturer’s instructions, cultivated for another 24 hours and fixed followed by ILEI immunostaining. Cells were fixed and permeabilized with 4% formaldehyde/0.1% Triton X-100 or with methanol:acetone (1:1) (for TGN38 staining). Immunofluorescence was detected by confocal microscopy (LSM 510/Axiovert 200 M (Zeiss, Oberkochen, Germany) and Leica SP5 (Leica Microsystems, Wetzlar, Germany)).

FLAG immunohistochemistry on paraformaldehyde-fixed, paraffin-embedded, 3-μm-thick sections of mouse tumor tissues was performed using the staining unit Discovery (Ventana Medical Systems, Tucson, AZ, USA).

### Experimental tumor and metastasis assays

Animal work was done by following earlier protocols that received ethical approval from the Institutional Animal Care and Use Committee of the Research Institute of Molecular Pathology and from the Austrian Bundesministerium für Bildung, Wissenschaft und Kultur. Tumor formation and metastatic ability of derivatives of EpC40 and 4T1 cells were determined by mammary gland fat pad and tail vein injections into 8- to 12-week-old NMRI athymic nude female mice (Charles River Wiga GmbH, Sulzfeld, Germany) as described previously [29],[31]. Each experimental group contained five to ten mice. For EpC40 tumor formation assays, 2 × 10^5^ cells were injected and tumor growth was monitored by weekly measurement of the tumor diameter. Tumor volume was calculated by the formula *a* × *b*^2^/2, with *a* representing the major and *b* the minor tumor diameter. For metastasis assays, 2 × 10^5^ recultivated, EpC40-derived tumor cells were injected into the tail vein. Aprotinin treatment of mice was carried out by a daily intraperitoneal injection of 4,000 kallikrein inactivator units (KIU) of aprotinin (Lactan) dissolved in 100 μl of sterile, filtered 0.9% NaCl for the entire period of the experiment.

Quantification of lung metastasis was performed by calculating the percentage of metastatic lung area. Each lung was cut through serially in 3-μm-thick sections at 300-μm intervals, sections were stained with hematoxylin and eosin and scanned (Mirax scanner; Zeiss). The absolute value of metastatic and total lung area of each section was determined using an algorithm developed in and executed using the Definiens software suite (Definiens, Munich, Germany), and the percentage of metastatic lung area per mouse was calculated from the sum of metastatic and total lung areas of all sections of the lung.

Metastasis analysis of derivatives of 4T1 cells was performed by injection of 5 × 10^5^ cells into the fifth pair of mammary gland fat pads. Mice were killed 30 days after injection, and primary tumors and lung metastases were analyzed as described above.

### Human breast cancer tissue array

All human tissue material was collected from patients who had undergone surgery at the Medical University of Vienna and was analyzed under protocol 141/2002 approved by the Institutional Review Board (IRB, “Ethikkommission”) of the Medical University of Vienna. Specific informed consent to participate in the study and to use samples and records was obtained from patients recruited after the onset of the study. For patients who underwent surgery before the start of the study, the IRB approved a waiver of specific informed consent. Two tissue arrays from 108 female breast cancer patients were analyzed retrospectively [7],[36]. They were processed manually for immunohistochemistry (IHC) using ILEI and uPAR (Santa Cruz Biotechnology, Santa Cruz, CA, USA) antibodies, and immunostained arrays were scanned in the Mirax scanner. Subcellular ILEI localization was scored as described elsewhere [7]. For tumor cell–associated uPAR expression a semiquantitative intensity score was used on a scale of 0 to 5 (0, no immunoreactivity; 1, very weak; 2, weak; 3 moderate; 4 strong; 5 very strong). No intensity and weak staining intensity (scores 0 to 2) were considered low uPAR expression, and moderate and strong staining intensities (scores >2) were considered high uPAR expression. This uPAR cutoff was validated with a receiver operating characteristic curve. ILEI scoring was performed by three independent observers and uPAR by two. For technical reasons, the ILEI IHC score was not evaluable for 12 patients, the uPAR score for 2 patients and both markers for 6 patients. Accordingly, all further analyses were based on 88 patients.

### Statistical analysis

Data are expressed as the mean ± SEM where applicable. Statistical significance was determined by unpaired, two-sided Student’s *t*-test. *P* < 0.05 was considered significant, and these values are marked with asterisks (*P < 0.05; **P < 0.01). Kaplan-Meier plots were computed with the survival package of R statistical software (The R Project for Statistical Computing, Vienna, Austria). Corresponding *P*-values were calculated as described elsewhere [37]. Correlations of ILEI and uPAR IHC status with clinical and histopathological parameters were evaluated with Fisher’s exact test.

## Results

### ILEI is processed extracellularly by the serine proteases plasmin, plasma kallikrein and neutrophil elastase

**Figure 1:**
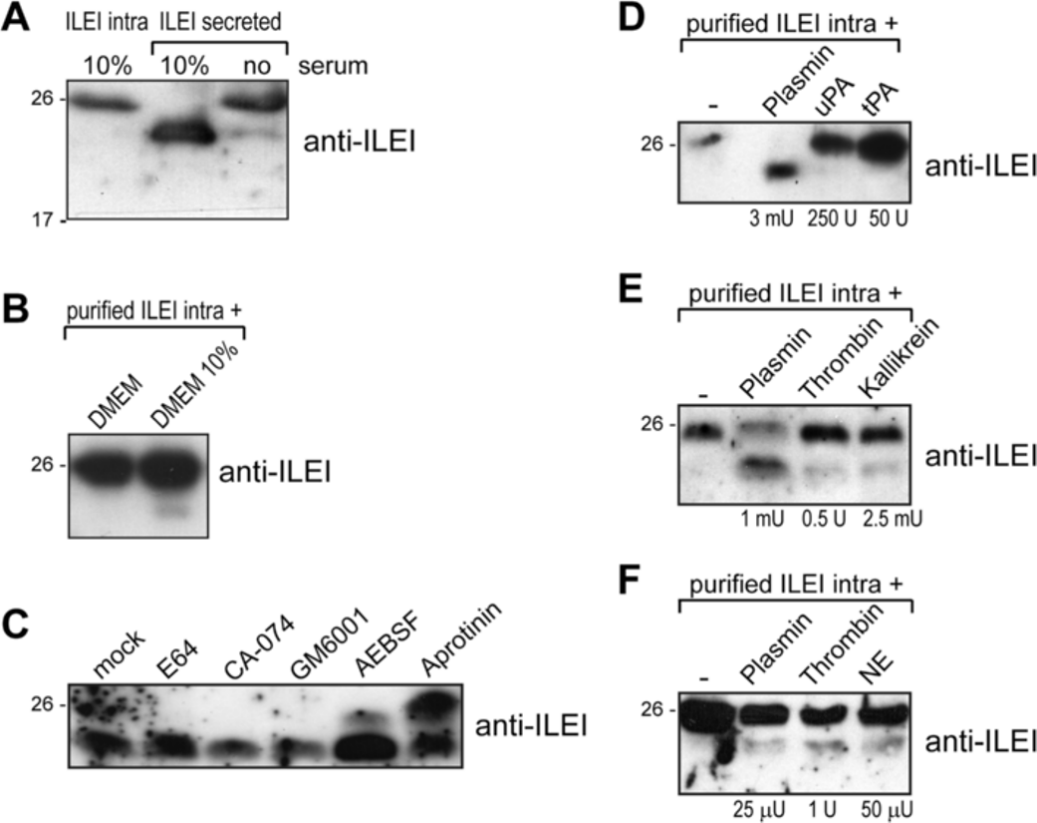
**Interleukin-like epithelial-to-mesenchymal transition inducer is processed extracellularly by serine proteases, most efficiently by plasmin. (A)** Western blot analysis of interleukin-like epithelial-to-mesenchymal transition inducer (ILEI) in whole-cell lysate and conditioned medium (CM) of EpRas cells grown in 10% fetal calf serum (FCS) or under serum-free conditions. Loading was not normalized to cell numbers. Full-length ILEI runs at the size of 26 kDa, the processed form approximately 2 kDA lower. **(B)** Purified full-length ILEI-6xHis was incubated with serum-free Dulbecco’s modified Eagle’s medium (DMEM) or medium containing 10% FCS (DMEM 10%) for 5 hours. ILEI protein was repurified via its 6xHis affinity tag and subjected to Western blot analysis. **(C)** Western blot analysis of ILEI in CM of EpRas cells cultured in the presence of the broad-specificity inhibitors against cysteine proteases (E64), cathepsin B (CA-074), matrix metalloproteases (GM6001) and serine-type proteases (4-(2-aminoethyl)-benzolsulfonylfluorid-hydrochloride (AEBSF) and aprotinin) for 24 hours. **(D)** to **(F)** Purified intracellular, full-length ILEI-6xHis was subjected to Western blot analysis after incubation for 2 to 5 hours with the indicated amounts of purified **(D)** plasmin, uPA and tPA; **(E)** plasmin, thrombin and plasma kallikrein; and **(F)** plasmin, thrombin and neutrophil elastase (NE).

Mass spectrometric analysis has shown that, in addition to the signal peptide, secreted ILEI lacks 17 amino acids (propeptide) at the N terminus, indicating a proteolytic processing of the protein [7]. Western blot analysis of whole-cell extracts and CM of EpRas cells, grown under normal and serum-free conditions, indicated that cleavage might occur in the extracellular space and pointed to an involvement of the serum in ILEI processing (Figure 1A). Importantly, purified ILEI was also cleaved by medium supplemented with 10% FCS (Figure 1B), albeit to a modest extent in comparison to the endogenously secreted protein. Neither secreted nor membrane-tethered components significantly increased the moderate ILEI-processing capacity of FCS in purified material (Additional file 1: Figure S1A), indicating that it might be a consequence of either the purification procedure or the influence of spatial arrangement of extracellular ILEI on cleavage. Solely inhibitors of serine proteases—aprotinin and, to a weaker extent, AEBSF—were capable of inhibiting ILEI cleavage in the CM of EpRas cells (Figure 1C), indicating that serine-type serum proteases are involved in ILEI processing.

To identify the ILEI-processing protease, we performed *in vitro* cleavage assays with candidate proteases. On the basis of their important role in cancer progression and in the processing of growth factors, we selected plasmin, tPA, uPA and NE [26],[38],[39]. However, we knew that cleavage should occur between arginine and serine residues. Thus, we also focused also on proteases known to have this consensus cleavage recognition site. These proteases were studied in addition to plasmin, thrombin and plasma kallikrein. Whereas tPA and uPA showed no activity, plasmin cleaved ILEI efficiently (Figure 1D). Thrombin, plasma kallikrein and NE were also able to process purified, full-length ILEI (Figure 1E and F). None of the enzymes acted on the short, processed form of the protein (data not shown), indicating that all of them recognized a cleavage site that led to the removal of the propeptide. Considering the very different amounts of these four proteases required for ILEI-processing plasmin, plasma kallikrein and NE were physiologically relevant candidates for ILEI cleavage.

To test the prevalent role of plasmin in ILEI processing, we analyzed ILEI in the CM of EpRas cells upon cultivation in Plg-depleted FCS. Plg depletion did not completely eliminate ILEI cleavage (Additional file 1: Figure S1B). In a further test, an ILEI cleavage assay was performed with blood plasma of *Plg*-KO mice. Similarly, lack of plasmin in the plasma only partially reduced the accumulation of processed ILEI (Additional file 1: Figure S1C), suggesting that other serine-type proteases, most probably NE and kallikrein, also act on ILEI at physiological concentrations.

### Identification of a physiological proteolytic cleavage site in ILEI

**Figure 2:**
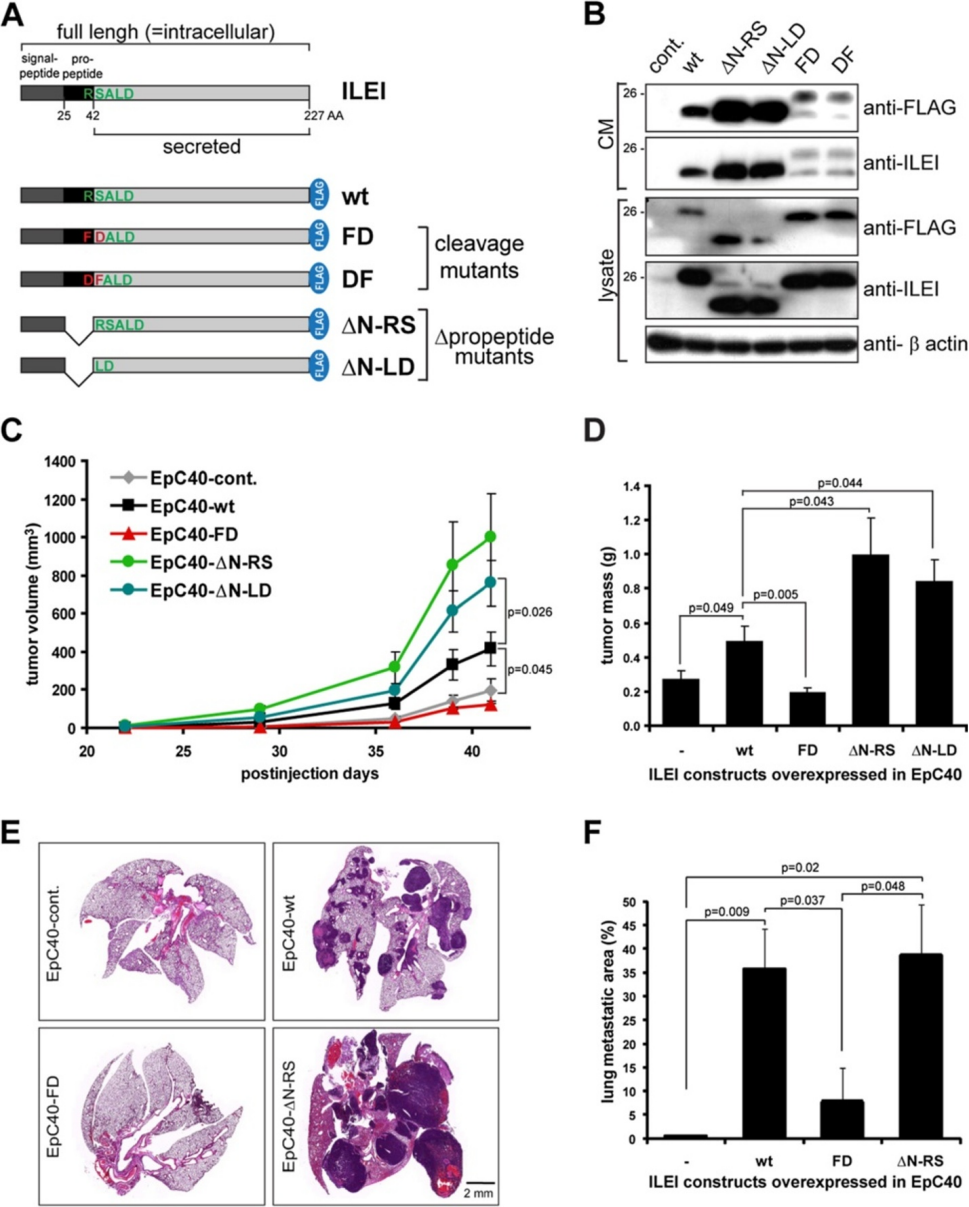
**Removal of the propeptide is required for elevated tumor growth and lung colonization of EpC40 cells. (A)** Schematic drawing of the interleukin-like epithelial-to-mesenchymal transition inducer (ILEI) protein and ILEI constructs used in this study. wt, Wild type. **(B)** ILEI and FLAG Western blot analysis of whole-cell lysates and conditioned medium (CM) of EpC40 cells overexpressing the ILEI constructs listed in **(A)**. **(C)** and **(D)** Primary tumor growth capacity of EpC40 cells overexpressing empty vector or different ILEI forms was determined upon injection into the mammary gland fat pads of female nude mice (*n* = 5 per group). **(C)** Tumor growth rate by calculated tumor volume 41 days after injection (±SEM). **(D)** Tumor mass 41 days after injection (±SEM). **(E)** and **(F)** Lung colonization capacity of EpC40 cells overexpressing empty vector or different ILEI forms was determined upon intravenous injection of recultivated cells of mammary tumors into female nude mice (*n* = 5 per group). Lungs were dissected and analyzed for metastases 45 days after injection. **(E)** Hematoxylin and eosin–stained histological sections of representative lungs of each group. Scale bar, 2 mm. **(F)** The percentage of lung metastatic area of each group (±SEM).

To validate the recognition site for proteolytic cleavage, mutations were introduced at the mapped N-terminal start of secreted ILEI generating the constructs FD and DF (cleavage mutants, Figure 2A). We also generated the ILEI constructs ΔN-RS and ΔN-LD lacking the propeptide sequence (Δ-propeptide mutants, Figure 2A). A lentiviral vector was used to introduce C-terminal, FLAG-tagged versions of these constructs and to generate stably infected EpC40 cells. The cleavage-mutant ILEI forms FD and DF were not cleaved by plasmin *in vitro* (Additional file 1: Figure S1D), showing that the mapped arginine-serine site is the cleavage recognition site.

Western blot analysis of whole-cell extracts verified that both cleavage-mutant and both Δ-propeptide-mutant forms of ILEI were efficiently expressed and secreted in EpC40 cells at their expected sizes, albeit to somewhat different levels (Figure 2B). Nevertheless, a weak band at the size of processed ILEI was observed in the CM of cleavage mutants. We regard it unlikely that the cleaving enzymes would still recognize the altered protein sequence. We rather would think that a second arginine-serine site, only six amino acids further toward the C terminus of the protein, might be recognized in these mutants. However, our mass spectrometric data indicate that the second cleavage site was not used under physiological conditions.

### Removal of ILEI propeptide is essential for ILEI-dependent increase in tumor growth and metastasis induction

To study the role of ILEI cleavage in tumor development, we tested the activity of the different ILEI mutants in tumor formation and metastasis assays. Mammary gland fat pad injections were performed with EpC40 cells overexpressing WT and mutant forms of the protein. As described elsewhere [7] and in Figure 2C and D, ILEI-wt-overexpressing EpC40 cells show significantly elevated tumor growth. Cleavage-mutant (FD) ILEI-overexpressing cells had low tumor growth capacity, similar to control cells (Figure 2C and D). Interestingly, the two Δ-propeptide ILEI forms (ΔN-RS and ΔN-LD) accelerated tumor growth of EpC40 cells even more than ILEI-wt did (Figure 2C and D). These data show that a noncleavable ILEI form had no activity, indicating that cleavage plays an important role in the activation of the protein. In addition, the moderate tumor growth capacity of ILEI-wt- compared to Δ-propeptide ILEI–overexpressing cells suggests that the mere availability of ILEI-processing proteases in the tumor microenvironment might be a regulatory factor of active ILEI levels.

Because EpC40 cells are not able to metastasize from primary tumor sites, recultivated cells of the above-described EpC40 tumors were injected intravenously into mice to model late aspects of metastasis. ILEI-wt- and Δ-propeptide ILEI–overexpressing tumor cells formed large lung metastases in all animals, as expected ([7] and Figure 2E and F). In contrast to primary tumor formation ability, the difference in the metastatic capacity between these cells was not significant. Cleavage-mutant, ILEI-overexpressing tumor cells showed only some weak capacity to induce small metastases (Figure 2E and F).

We tested the general necessity of proteolytic processing for ILEI function by analyzing the activity of the different ILEI forms in metastatic 4T1 murine mammary cancer cells that expressed and secreted high levels of ILEI, too (Additional file 2: Figure S2A). Whereas EMT/metastasis promoter and suppressor genes also influence primary tumor growth in EpC40 cells [5],[7],[40], they act exclusively on the metastatic capacity of 4T1 cells from the orthotopic site [31],[41],[42]. Consistent with this, stable RNAi-KD of ILEI solely decreased lung metastasis of 4T1 cells (Additional file 2: Figures S2B to S2D). The metastatic behavior of the different ILEI forms was tested by reconstituting 4T1 ILEI-KD cells with the above-described ILEI constructs (Additional file 2: Figure S2A). Importantly, reexpression of WT and Δ-propeptide (ΔN-RS) ILEI turned these cells highly metastatic again in a comparable manner, whereas cleavage mutant (FD) ILEI showed only weak metastasis-promoting activity (Additional file 2: Figures S2C and S2D). None of the reexpressed ILEI forms had a significant effect on primary tumor size (Additional file 2: Figure S2B). In summary, the metastatic capacity of 4T1 ILEI-KD cells reconstituted with different mutant ILEI forms was reminiscent of the lung colonization activity of these proteins upon overexpression in EpC40 cells, indicating that our observations in EpC40 cells may have general validity, in breast cancer cells at least.

### Inhibition of ILEI processing interferes with elevated tumor growth and metastasis induction

**Figure 3:**
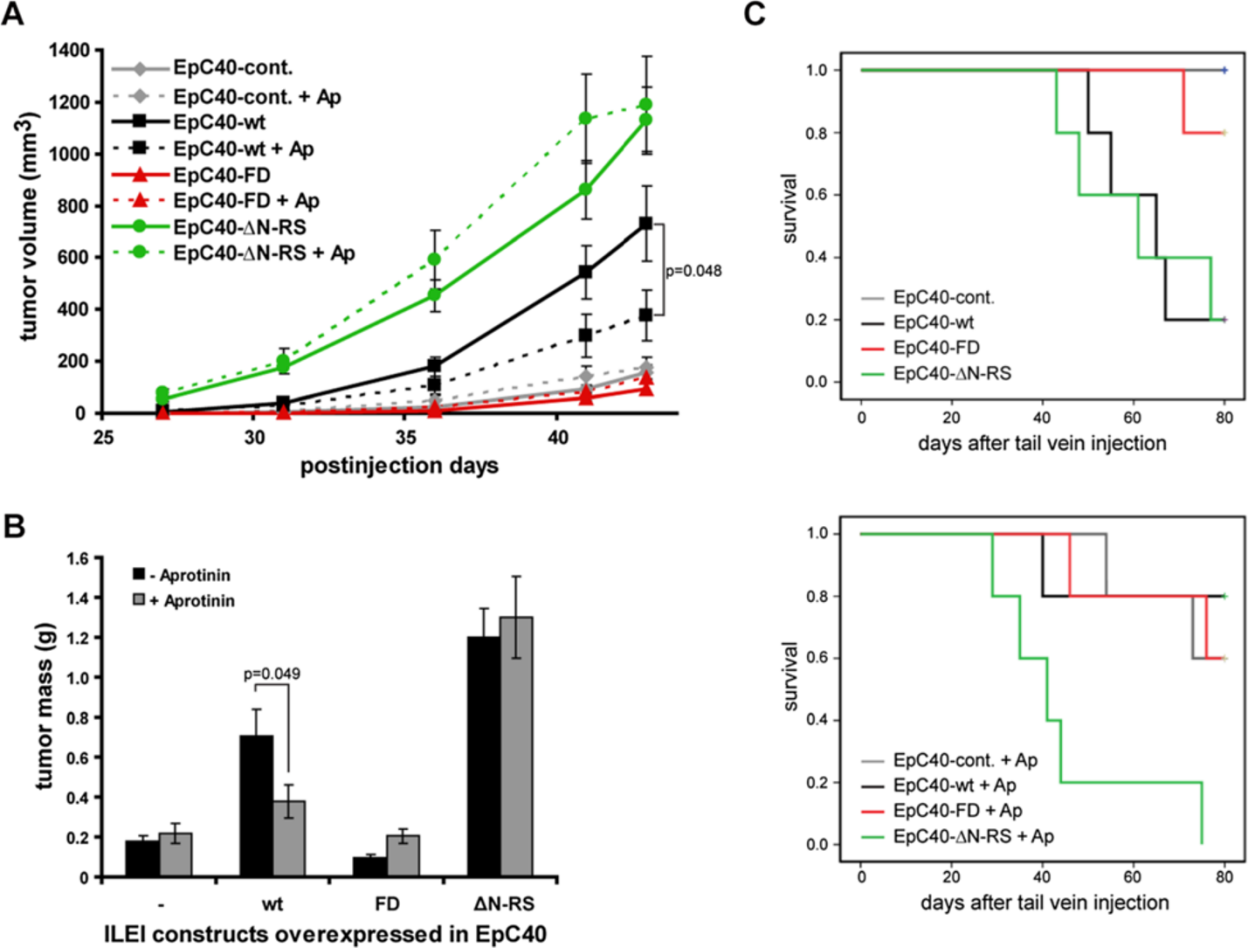
**Aprotinin suppresses increased tumor growth and lung colonization of wild-type interleukin-like epithelial-to-mesenchymal transition inducer**–**overexpressing EpC40 cells.** Analysis of primary tumor growth and lung colonization capacity of EpC40 cells and derivatives in aprotinin (Ap)-treated mice was performed as described in the Figure 2 legend (*n* = 10 per group). Five mice in each group were administered with 4,000 kallikrein inactivator units of aprotinin daily. Error bars show mean ± SEM. **(A)** Tumor growth rate. **(B)** Tumor masses. **(C)** Lung colonization assay. Moribund mice were killed and analyzed for lung metastases. The experiment was terminated 80 days after tumor cell injection. The frequency of survival is represented in two separate Kaplan-Meier plots, with the upper showing untreated mice and the lower aprotinin-treated mice.

To investigate the necessity of ILEI processing for both increased tumor growth and metastasis formation, we retested the tumorigenic and lung colonization ability of EpC40 cells overexpressing different ILEI forms in aprotinin-treated mice. Importantly, the ILEI-induced increase in tumor growth was suppressed by aprotinin treatment of ILEI-wt-overexpressing cells, but not Δ-propeptide ILEI–overexpressing cells (Figure 3A and B), indicating that serine protease activity was essential to generate the processed functional form of ILEI *in vivo*.

Aprotinin is known to accelerate metastasis formation due to its effect on blood clotting [43]. Accordingly, all tumor cell lines showed a certain capacity in the lung colonization assay to induce metastasis-driven terminal disease upon aprotinin treatment, though with very different kinetics. Aprotinin accelerated the onset of terminal sickness of mice injected with Δ-propeptide (ΔN-RS) ILEI-overexpressing cells by about 2 weeks and moderately decreased survival of mice injected with cleavage-mutant (FD) ILEI-overexpressing or control EpC40 cells (Figure 3C). Most importantly, however, ILEI-wt-overexpressing tumor cells showed a significant decrease in metastatic capacity in aprotinin-treated mice (Figure 3C). These data show that aprotinin decreased metastatic capacity only in cells that were sensitive for ILEI processing, indicating that proteolytic processing of ILEI is also essential for metastasis formation. Hence, the differences in the activity of the different ILEI forms in tumorigenesis and metastasis assays are most likely a consequence of saturating ILEI-processing protease activity in blood and lung compared to tumor tissue. Because cleavage-mutant ILEI was still a weak substrate for processing, its residual metastatic capacity might also be explainable by high local protease concentrations.

### Propeptide tethers ILEI to fibronectin-containing extracellular matrix complexes

**Figure 4:**
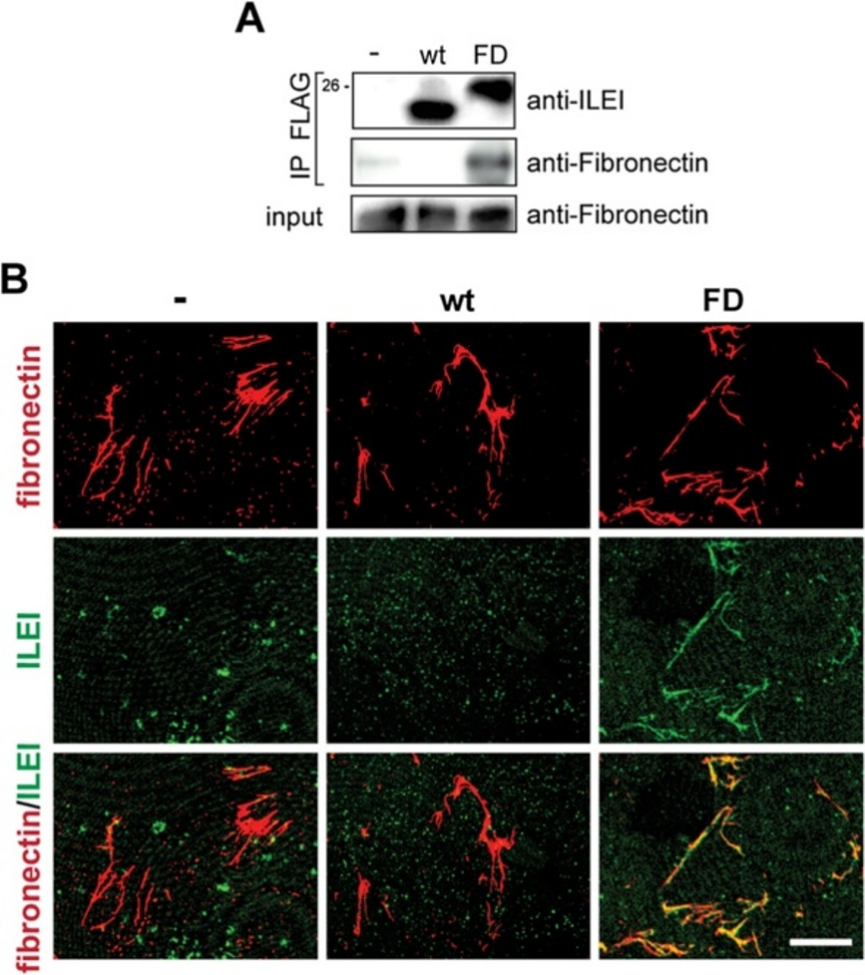
**Interleukin-like epithelial-to-mesenchymal transition inducer binds to soluble and deposited fibronectin-containing extracellular matrix complexes via its propeptide. (A)** Western blot analysis of wild-type (wt) or cleavage mutant (FD) interleukin-like epithelial-to-mesenchymal transition inducer (ILEI) immunoprecipitated (IP) from conditioned medium of overexpressing EpRas cells via their FLAG epitope tags. Coprecipitation of extracellular soluble fibronectin was probed using a fibronectin-specific antibody. **(B)** Immunofluorescence analysis of purified WT and cleavage mutant (FD) ILEI-6xHis proteins after incubation with prepared cell-free extracellular matrix (ECM) deposited by NIH 3T3 fibroblasts. Bound ILEI proteins were visualized via the 6xHis affinity tag using penta-His antibody (green). ECM was counterstained with a fibronectin-specific antibody (red). The purification fraction of empty vector expressing EpRas cells was used as control. Scale bar, 5 μm.

To investigate the molecular role of ILEI processing, we aimed to find interactors specifically binding processed or nonprocessed ILEI. For this purpose, FLAG immunoprecipitates of WT and cleavage-mutant (FD) ILEI from CM of overexpressing EpRas cells were analyzed by mass spectrometry. We identified fibronectin in the precipitate of cleavage-mutant ILEI (FD), but not ILEI-wt. This interaction was confirmed in coimmunoprecipitation experiments using the same conditions as we employed for mass spectrometry. Fibronectin efficiently coprecipitated only with cleavage-mutant (FD) ILEI (Figure 4A), suggesting that ILEI interacts with soluble fibronectin in a propeptide-dependent manner.

Fibronectin is a component of the ECM. This prompted us to test whether ILEI would be able to bind to the ECM in a propeptide-dependent manner. Purified, processed WT and cleavage-mutant (FD) 6xHis-tagged ILEI were incubated with prepared cell-free ECM deposited by NIH 3T3 fibroblasts. Only cleavage-mutant (FD), and not processed, ILEI-wt accumulated along deposited fibers of the ECM, which we visualized by using a fibronectin-specific antibody (Figure 4B). These data are consistent with our immunoprecipitation results and support the idea that the ILEI propeptide plays an important role in tethering the protein to the ECM by direct or indirect interaction with fibronectin and that release may occur by proteolytic cleavage.

### Plasmin induces ILEI secretion by rapidly mobilizing its intracellular protein pool

**Figure 5:**
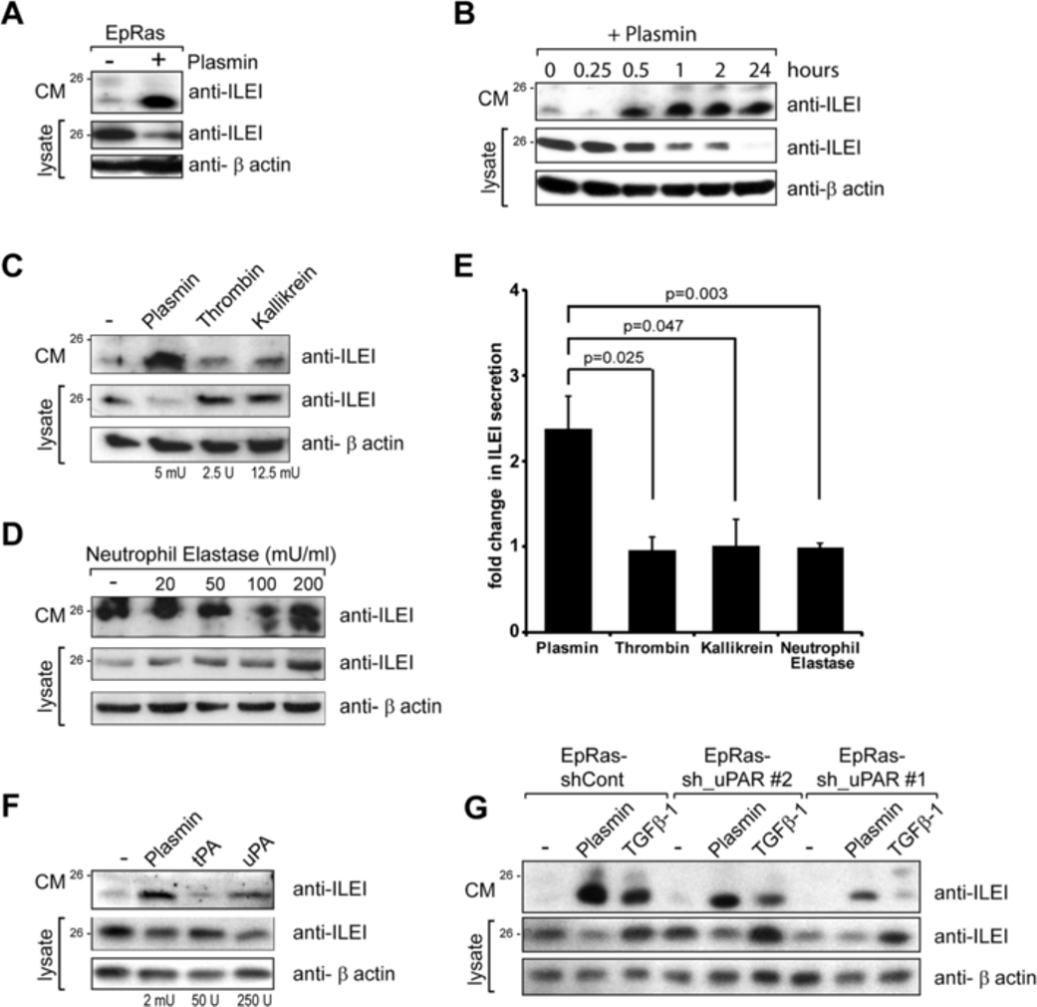
**Plasminogen-urokinase plasminogen activator receptor signaling rapidly upregulates interleukin-like epithelial-to-mesenchymal transition inducer secretion by mobilizing its intracellular protein pool. (A)** to **(D)** Western blot analysis of interleukin-like epithelial-to-mesenchymal transition inducer (ILEI) in whole-cell lysates and conditioned medium (CM) of EpRas cells not treated or treated with plasmin for 16 hours after serum withdrawal **(A)**, harvested 24 hours after serum withdrawal and incubation with plasmin for the indicated periods of time **(B)**, treated with indicated concentrations of plasmin, thrombin or plasma kallikrein for 24 hours after serum withdrawal **(C)** and treated with indicated concentrations of neutrophil elastase (NE) for 24 hours after serum withdrawal **(D). (E)** Fold change ± SEM in ILEI secretion of EpRas cells after plasmin, thrombin, plasma kallikrein and NE treatments calculated by semiquantification of Western blots of three independent experiments. **(F)** and **(G)** Western blot analysis of ILEI in whole-cell lysates and CM of EpRas cells treated with indicated concentrations of plasmin, tissue plasminogen activator (tPA) or urokinase plasminogen activator (uPA) for 24 hours after serum withdrawal **(F)** and in control and urokinase plasminogen activator receptor (uPAR) short-hairpin RNA EpRas cells not treated or treated with plasmin or transforming growth factor β-1 (TGFβ-1) for 24 hours after serum withdrawal **(G)**.

We confirmed that plasmin was sufficient to process secreted ILEI in EpRas cell culture. More strikingly, however, a dramatic increase in ILEI secretion of these cells was observed upon plasmin treatment, combined with a decrease in the intracellular levels of the protein (Figure 5A). We tested the dose dependency of plasmin on these effects (Additional file 3: Figure S3A). The lowest amount of plasmin was already able to fully stimulate ILEI secretion, whereas nonprocessed ILEI disappeared only with increasing concentrations of plasmin, suggesting that secreted ILEI might primarily accumulate in its uncleaved, inactive form at limiting concentrations of plasmin.

Plasmin appeared to be even more potent in increasing ILEI secretion than TGFβ (Additional file 3: Figure S3B). ILEI secretion reached maximal levels as soon as 1 hour after addition of plasmin, combined with a significant decrease in the intracellular level of the protein (Figure 5B). In contrast, TGFβ upregulated intracellular ILEI within 4 to 8 hours via its effect on translation [7],[10]; however, secretion levels increased only after 8 hours (Additional file 3: Figure S3C). In summary, plasmin, unlike TGFβ, rapidly stimulated ILEI secretion at the expense of the intracellular ILEI protein pool.

### Plasminogen-urokinase plasminogen activator receptor signaling regulates ILEI secretion

To discover whether the effect of plasmin on ILEI secretion depended on ILEI cleavage, we tested the capability of the other ILEI-processing proteases—thrombin, kallikrein and NE—to enhance secretion of the protein. In contrast to plasmin, they neither increased secreted intracellular ILEI levels nor decreased them in EpRas cells (Figure 5C to E), suggesting that plasmin might induce secretion independently of ILEI cleavage.

We therefore focused on the potential role of uPAR signaling in ILEI secretion. We tested whether direct activation of uPAR could induce ILEI secretion. Incubation of EpRas cells with active uPA induced ILEI secretion in a dose-dependent manner, and it was coupled with a decrease in intracellular ILEI levels (Figure 5F and Additional file 3: Figure S3D). To show directly that uPAR is essential in the regulation of ILEI secretion, we generated stable uPAR-KD EpRas cells and tested them for their capability to induce ILEI secretion upon plasmin treatment. EpRas sh_uPAR-1 cells showed efficient depletion (approximately 80%; see Additional file 3: Figure S3E), and EpRas sh_uPAR-2 showed moderate depletion, of uPAR expression (about 50%; see Additional file 3: Figure S3E). Importantly, plasmin-induced ILEI secretion was significantly impaired in these cells and correlated with their residual amount of uPAR in a dose-dependent manner (Figure 5G), indicating that uPAR is required in this process. In accordance with this finding, intracellular ILEI levels of these cells either were not or were only moderately decreased (Figure 5G). Depending on the culture conditions, EpRas cells with partial uPAR KD (EpRas-sh_uPAR-2) showed differences in their sensitivity to plasmin-induced ILEI secretion (data not shown). Interestingly, uPAR KD not only interfered with plasmin-induced ILEI secretion but also inhibited TGFβ-induced secretion (Figure 5G), indicating that uPAR might be a more general regulator of ILEI secretion by integrating several signals. In summary, these results strongly support the hypothesis that plasmin-induced upregulation of ILEI secretion is independent of its activity on ILEI cleavage and acts most probably via uPAR signaling. In addition, although TGFβ- and plasmin-induced ILEI secretion could be uncoupled at the level of the translational control of ILEI, they might share the same uPAR-dependent regulatory mechanism of secretion.

We also analyzed the uPAR-dependent regulation of ILEI secretion in a series of human breast cancer cell lines. Whereas nonmetastatic cell lines (T47D and MCF7) expressed and secreted low levels of ILEI and did not express uPAR, uPA, tPA PAI-1 or PAI-2, metastatic cell lines (MDA-MB-468, MDA-MB-231 and CAMA1) expressed and secreted high ILEI levels and expressed uPAR and partially expressed additional components (Additional file 4: Figures S4A and S4B). Furthermore, plasmin treatment of MCF7 and MDA-MB-231 cells induced increased ILEI secretion only in uPAR-expressing MDA-MB-231 cells (Additional file 4: Figure S4C). In summary, the extent and inducibility of ILEI secretion correlated with the expression levels of uPAR in these cells, indicating that the necessity of uPAR signaling for ILEI secretion might also be relevant in humans.

### ILEI secretion levels correlate with altered subcellular localization of the protein

**Figure 6:**
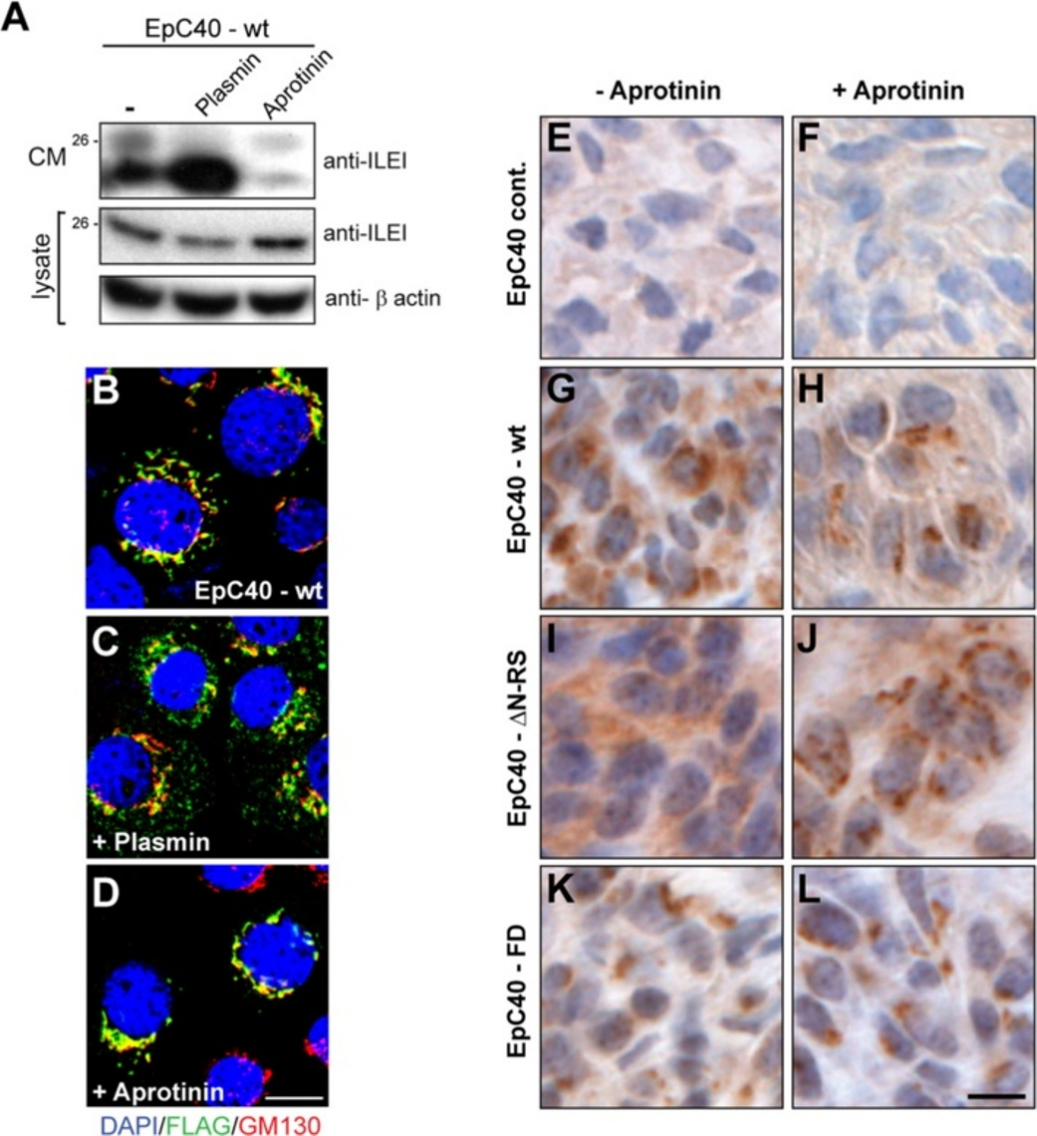
**Interleukin-like epithelial-to-mesenchymal transition inducer secretion levels correlate with altered subcellular localization of the protein. (A)** Western blot analysis of interleukin-like epithelial-to-mesenchymal transition inducer (ILEI) in whole-cell lysates and conditioned medium (CM) of wild-type ILEI-overexpressing EpC40 cells (EpC40-wt) harvested 32 hours after serum withdrawal with or without treatment with plasmin (10 mU/ml) for 8 hours before harvest or aprotinin (10 μM) for 32 hours before harvest. CM of the last 24 hours were collected. **(B)** to **(D)** Immunofluorescence analysis of EpC40-wt cells as described in **(A)** without treatment **(B)** or after plasmin treatment **(C)** or aprotinin treatment **(D)**. ILEI is visualized via its FLAG tag (green), Golgi is marked by a GM130 antibody (red) and genomic DNA is counterstained with DAPI (4′,6-diamidino-2-phenylindole, blue). Scale bar, 10 μm. **(E)** to **(L)** Immunohistochemistry for ILEI localization on representative 3-μm-thick sections of paraffin-embedded EpC40 mammary tumors (*n* = 5 per group) overexpressing the empty vector control (cont.; (**E**) and (**F**)) or the following ILEI constructs: EpC40-wt **(G)** and **(H)**, EpC40-ΔN-RS **(I)** and **(J)** and EpC40-FD **(K)** and **(L)**. **(E, G, I and K)** Tumors from nontreated animals. **(F, H, J and L)** tumors from aprotinin-treated animals. Overexpressed ILEI is shown via its FLAG epitope tag (brown), and genomic DNA is counterstained with hematoxylin (blue). Scale bar, 10 μm.

Altered subcellular localization of ILEI correlates with metastasis-free survival in breast cancer, and ILEI secretion correlates with the metastatic capacity of several cancer cell lines [7]. This prompted us to investigate whether differences in ILEI secretion levels would be reflected in an altered subcellular localization of the protein. For this purpose, we induced elevated or decreased ILEI secretion in ILEI-wt-overexpressing EpC40 cells by plasmin or aprotinin treatment (Figure 6A) and analyzed the subcellular localization of the protein. Normally, ILEI localized to the Golgi and trans-Golgi networks (TGNs) and formed additional dotted structures in the cytoplasm (Figure 6B and Additional file 5: Figure S5). The latter ones colocalized with neither endosomal and lysosomal organelle markers nor Rab8a (Additional file 5: Figure S5), indicating that they most likely represent secretory vesicles independent of the endosomal recycling and basolateral sorting routes [44],[45]. Plasmin treatment triggered a remarkable expansion of vesicular ILEI staining (Figure 6C), whereas the addition of aprotinin restricted ILEI to Golgi structures (Figure 6D). This indicates that elevated ILEI secretion might be linked to the accumulation of ILEI secretory vesicles throughout the cytoplasm.

To see how these different subcellular ILEI localizations would be discernible in tumor tissue, we performed immunohistological analysis of overexpressed ILEI forms in EpC40 tumors of non-treated and aprotinin-treated mice (Figure 6 E to L). Aprotinin efficiently inhibited ILEI-processing proteases in these tumors, implicating that secretion was also affected. Consistently, aprotinin treatment shifted large parts of the ILEI signal to strongly stained granules in both WT and Δ-propeptide (ΔN-RS) ILEI–overexpressing tumor cells (Figure 6G J). Cleavage-mutant (FD) ILEI preferentially localized to dense granules independently of aprotinin (Figure 6K and L).

In summary, these results suggest that the subcellular localization of ILEI correlates with the secretory status of the protein, with granular staining corresponding to tubular Golgi and TGN structures and to low levels of ILEI secretion, and with diffuse cytoplasmic staining reflecting dispersed secretory vesicles and high ILEI secretion levels.

### Cytoplasmic ILEI localization strongly correlates with high uPAR expression and poor prognosis in human breast cancer

**Figure 7:**
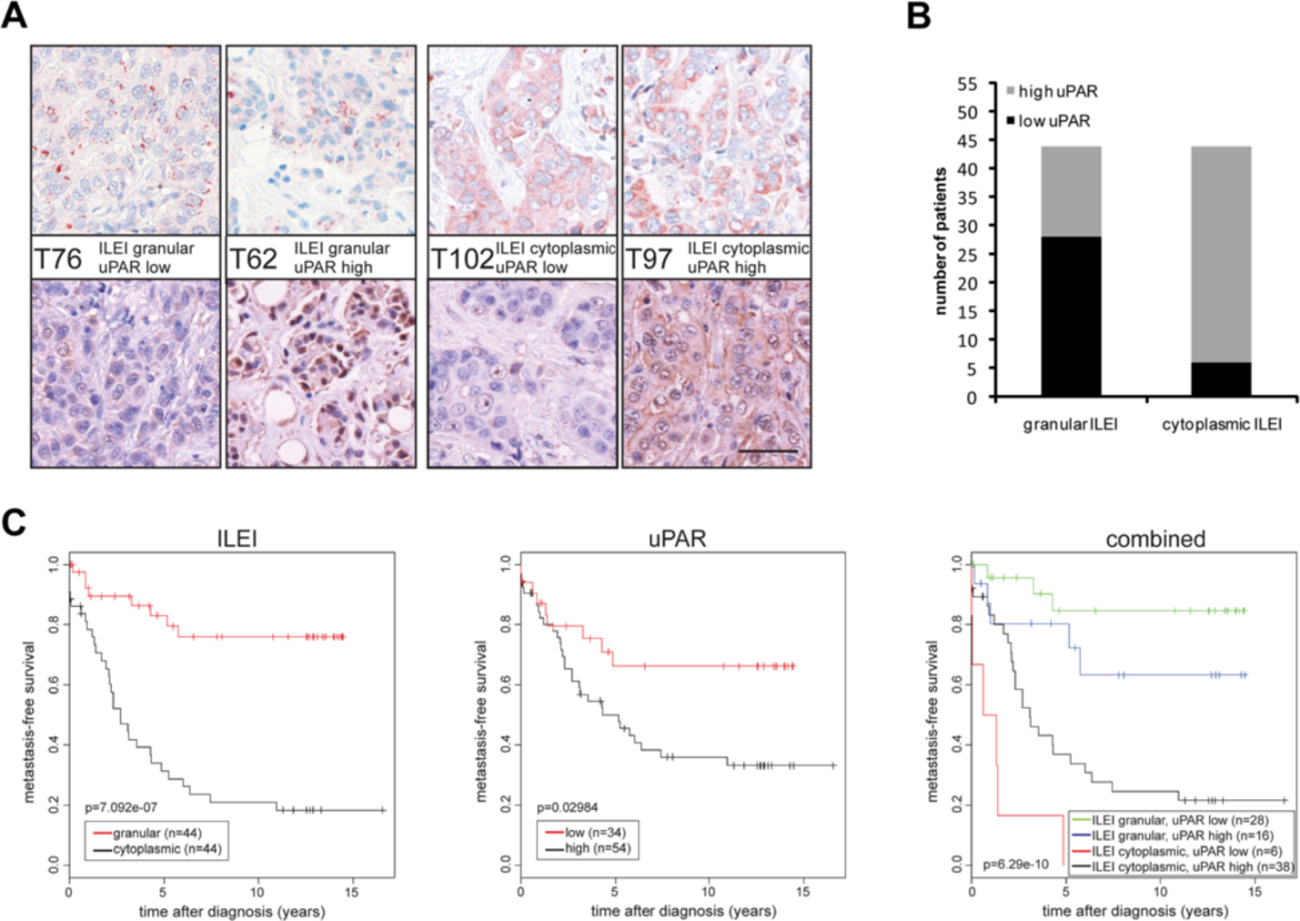
**Cytoplasmic interleukin-like epithelial-to-mesenchymal transition inducer localization correlates with high tumor-cell associated urokinase plasminogen activator receptor expression, and a combined ILEI-uPAR marker analysis shows enhanced prognostic power in human breast cancer. (A)** Immunohistochemistry for interleukin-like epithelial-to-mesenchymal transition inducer (ILEI) localization (granular or cytoplasmic, upper images) and urokinase plasminogen activator receptor (uPAR) expression (low or high, lower images) on representative samples from two human breast cancer tissue arrays. ILEI/uPAR signal is in purple, and genomic DNA is in blue (counterstained with hematoxylin and eosin). Scale bar, 50 μm. **(B)** Distribution of tumor-cell associated uPAR protein expression levels in 88 patients with different subcellular ILEI localizations. **(C)** Evaluation of ILEI localization (left panel), tumor-cell associated uPAR expression (middle panel) and a combined analysis of the two markers (right panel) for metastasis-free survival depicted in Kaplan-Meier plots.

To explore the relevance of our findings in human cancer pathogenesis, we analyzed the extent to which ILEI secretion, and thus subcellular localization of ILEI, correlated with tumor cell–associated uPAR protein expression in human breast tumor tissue samples. Accordingly, immunohistochemistry for ILEI and uPAR was performed on two human breast cancer arrays (Figure 7A). Whereas tumors with granular ILEI localization showed divergent uPAR levels, almost 90% of the carcinomas with cytoplasmic ILEI localization showed high uPAR expression (Figure 7B). These data reveal a tight correlation between cytoplasmic ILEI localization and high uPAR levels in human breast cancer and further confirm that uPAR expression is an important prerequisite of elevated ILEI secretion in tumor cells.

Both cytoplasmic ILEI localization and high tumor-cell associated uPAR expression correlated significantly with a shorter metastasis-free life span (Figure 7C, left and middle panel, and [7],[46],[47]). We tested if a combined Kaplan-Meier survival analysis of the two markers would provide an enhanced prognostic power (Figure 7C, right panel). In the combined analysis, the four categories distributed into clearly distinct survival frequencies (Figure 7C, right panel). The combination “granular ILEI, low uPAR” was able to identify patients with the best survival chances. High uPAR expression indicated a moderately but significantly decreased probability of survival of patients with granular ILEI localization. Cytoplasmic ILEI localization was almost exclusively coupled to high uPAR expression, thus the combined analysis gave survival frequencies similar to those of cytoplasmic ILEI alone. Surprisingly, the rare group “cytoplasmic ILEI, low uPAR” showed the lowest frequency of survival. However, the low incidence (6 of 88) and case number precluded conclusions about the biological relevance of this group.

Breast cancer is a heterogeneous disease [48]. To test if the prognostic effects of ILEI, uPAR and an ILEI-uPAR combined analysis were applicable to all breast cancers, ILEI and uPAR scores were correlated with the metastasis-free survival of patients divided into the four major breast cancer subtypes. These were defined by estrogen receptor (ER), progesterone receptor (PR) and human epidermal growth factor receptor 2 (HER2) expression as luminal A (ER + and/or PR+, HER2−), luminal B (ER + and/or PR+, HER2+), triple-negative (ER−, PR−, HER2−) and HER2 type (ER−, PR−, HER2+) [49],[50]. All four subtypes were present in the cohort of the eighty-eight analyzed patients, and metastasis-free survival significantly differed by subtype (Additional file 6: Figure S6A). Importantly, the ILEI, uPAR and combined categories distributed into distinct survival frequencies within the luminal A, triple-negative and HER2 subtypes (Additional file 6: Figure S6B). In addition, the relative course of the survival frequencies of the four ILEI-uPAR categories in all these subtypes was reminiscent of the bulk analysis of all breast cancer patients (compare Figure 7C and Additional file 6: Figure S6B, right panel). However, the low number of patients in each group precluded making any statistically reliable conclusions about a potentially enhanced prognostic power of these marker analyses over cancer subtyping.

The four categories of ILEI and uPAR scores were correlated with established clinical and histopathological classifications of human breast cancer. As previously found for ILEI localization [7], the combined ILEI-uPAR scores maintained a significant correlation exclusively with the HER2 status of tumors (Additional file 7: Table S1).

These analyses confirmed the strong independent prognostic validity of ILEI localization for cancer survival. Furthermore, they indicate a tight linkage of ILEI localization and tumor cell–associated uPAR expression, the combined analysis of which might be beneficial in prospectively identifying patients with the lowest, and possibly the highest, risk of metastasis in breast cancer.

## Discussion

We found plasmin, plasma kallikrein and NE to be able to cleave ILEI at physiological concentrations. As part of the blood coagulation–fibrinolytic system, both plasmin and kallikrein are present in the blood at high levels. The plasma concentration of NE is low in healthy conditions, but it is highly elevated during chronic inflammation, which is often linked to cancerous diseases [26]. Thus, as soon as tumor cells enter the circulation, the secreted ILEI protein is most likely processed and active. This is what our pulmonary metastasis studies indicate; however, as our tumor formation experiments show, the level of ILEI-processing proteases may be limited in the primary tumor, suggesting that they may have a decisive regulatory role at the site of tumor cell dissemination. Plasmin, kallikrein and NE are potentially present in the tumor stroma and could execute ILEI cleavage [14],[26],[51]. Plasma kallikrein could even play an enhancer function, because it can also activate Plg, uPA and the release of NE [52]. Hence, the relative abundance of these proteases might set the final level of processed ILEI in the primary tumor. The network of these proteases might also orchestrate ILEI secretion. We found that plasmin is a key factor in this process. Kallikrein is known to activate Plg and uPAR signaling [22], and NE is able to cleave uPAR and PAI-1 [53],[54]. Hence, both proteases could thereby influence the net intensity of uPAR-mediated ILEI secretion. In addition, plasmin, kallikreins and NE are proteases with ECM-degrading activity [26],[51] and thus might also contribute to increased ILEI activity by ECM degradation–driven release of the tethered protein. In summary, all three enzymes are good candidates to have important regulatory function on ILEI activity at multiple levels, such as regulated secretion and release from the ECM by direct proteolytic processing of the cytokine or by ECM degradation.

Plasmin had a very robust effect on ILEI secretion. In addition, active uPA was sufficient, and uPAR was essential, in the upregulation of ILEI secretion, suggesting a Plg–uPA–uPAR axis of the regulatory mechanism. Yet, there are numerous other ways that uPAR could be activated [19], and additional targets of plasmin might be relevant as well. The way in which uPAR increases ILEI secretion remains unclear, too. Thus, the identification of the relevant Plg–uPAR network and the uPAR coreceptor will be critical in deciphering the mechanism of uPAR-regulated ILEI secretion. In the present study, we observed that elevated ILEI was secreted within 30 minutes after plasmin treatment, suggesting that uPAR signaling primarily acts on the ILEI secretory pathway, most probably by regulating the budding of ILEI secretory vesicles from the TGN.

We demonstrate that plasmin is involved in the proteolytic cleavage of ILEI and is also responsible for the regulation of ILEI secretion. Thus, we expected protease inhibitor treatment to affect both processes. However, only cleavage-sensitive WT, and not Δ-propeptide ILEI, showed decreased tumor growth and metastasis following aprotinin treatment. Importantly, aprotinin inhibited ILEI secretion, and secretion of Δ-propeptide ILEI and ILEI-wt were similarly affected. This discrepancy can be explained by the fact that induction of ILEI secretion required lower plasmin levels than proteolytic cleavage. Thus, residual protease activity after aprotinin treatment might have been sufficient to maintain reasonable levels of ILEI secretion, but secreted ILEI-wt most probably remained unprocessed and inactive, whereas Δ-propeptide ILEI could function. These studies show that aprotinin-sensitive proteolytic processing plays an essential role in ILEI function and that plasmin-induced ILEI secretion is independent of this process. Nevertheless, overexpressed Δ-propeptide, WT and cleavage-mutant forms of ILEI showed differences in their secretion capacities that correlated with their localization patterns and activities. Although we could not detect differences in the expression levels of important components of the Plg–uPAR system in overexpressing cells, it is possible that autocrine signaling is involved in the feedback regulation of ILEI secretion.

In contrast to earlier findings, however, no evidence for an autocrine ILEI signaling was found during our *in vitro* analyses of the activity of the different ILEI mutants. We also found no differences in the anchorage-independent growth or in assays to address the role of EMT *in vitro* (three-dimensional cell culture assay and analysis of EMT markers) in the tumor cells overexpressing different ILEI forms compared to their controls (data not shown). Very importantly, and as a major difference from the earlier studies on ILEI, however, all experiments were performed with cell pools in our study. Thus, we did not perform any clonal selection of cells *in vitro* after introducing the different ILEI constructs; instead, clonal selection took place *in vivo* in the tumors. By this process, differences in the activity of the different ILEI forms might have been masked *in vitro* within the cell pools, but could be efficiently demonstrated *in vivo* in the correlative tumor formation and metastasis assays.

Our human breast cancer tissue array analysis showed an enhanced prognostic value of a combined ILEI-uPAR analysis. Granular ILEI localization coupled with high uPAR expression levels predicted somewhat lower survival frequencies than the combination of granular ILEI-low uPAR. A low ILEI secretion level despite high uPAR expression indicates other important components of uPAR signaling/ILEI secretory machinery missing in those tumors. Hence, a worse prognosis might be due to other known invasion- and metastasis-promoting actions of uPAR. Cytoplasmic ILEI was associated with poor prognosis and was coupled with high uPAR levels in almost 90% of the cases. The few cases showing cytoplasmic ILEI despite low uPAR levels, however, unexpectedly had the worst survival prognosis. Only an extended analysis of tumor samples will be able to answer the biological relevance of this group. Further experiments on a larger data set will be needed to show how other prognostic markers of the uPAR system (for example, uPA and PAI-1 [47],[55]) correlate with ILEI localization and whether high expression of these markers could compensate for low uPAR levels. The prognostic value of ILEI localization and of a combined ILEI-uPAR analysis was potentially relevant for all four major breast cancer subtypes that were investigated in this study. However, the low number of patients included is a clear limitation of the study, and a larger number of cases will be required for an accurate statistical analysis in the future. It will be also be interesting to see how the prognostic value of a combined ILEI-uPAR analysis correlates with other subtyping biomarkers that were not included in our present study, such as the basal markers epidermal growth factor receptor and cytokeratin 5/6 of triple-negative tumors.

## Conclusions

Our results suggest that plasmin plays a pivotal role in the regulation of ILEI processing and secretion and that uPAR-regulated ILEI secretion levels are decisive in tumor progression. They also indicate that the Plg-uPAR system may serve as a potential therapeutic target and an important prognostic factor in ILEI-dependent breast tumor progression.

## Authors’ contributions

AC developed the hypotheses and was a major contributor in designing and performing most of the experiments, analyzing the data and in writing the manuscript. BK participated in the design and carrying out of the experiments, including ILEI purification, cleavage and secretion assays and *in vivo* tumorigenesis and lung colonization assays with and without drug treatment. SW and US designed and generated lentiviral expression constructs and performed *in vivo* metastasis assays of 4T1 cells and tumor formation assays of EpC40 cells, respectively. SMM was involved in the design and execution of cleavage and secretion assays with Plg-depleted serum and NE and contributed to data analysis and manuscript editing. MS provided the breast cancer tissue arrays and performed clinical cohort data analysis. MA developed and performed immunohistochemistry and evaluated the resulting data and was involved in the execution and analysis of cell culture work and biochemical assays. KA developed a new algorithm for the quantification of lung metastases and coordinated the analysis of these data. GFV designed and performed the subcellular localization studies, including the generation of new subcellular marker constructs, in the laboratory of LAH. LAH provided key experimental reagents and advice, edited the manuscript and took up mentorship after the death of HB. HB contributed to the conception, design and coordination of the study until his death. All authors contributed to the drafting of the manuscript, read and approved the final manuscript, and agreed to be accountable for all aspects of the work.

## Additional files

## Electronic supplementary material

Additional file 1: Figure S1.: Loss of Plg partially impairs ILEI-processing capacity of serum and plasma, and mutation of the proteolytic cleavage site in ILEI prevents processing by plasmin. (A) Purified full-length ILEI-6xHis was incubated with medium containing 10% FCS (DMEM 10%) or CM of EpRas cells (EpRas CM) or added directly to the culture of EpRas cells (EpRas co-inc.) for 24 hours. ILEI protein was repurified via its 6xHis affinity tag and evaluated by Western blot analysis. (B) Western blot analysis of ILEI in CM of EpRas cells grown in the presence of 10% complete or Plg-depleted FCS. Loading was normalized to cell numbers. (C) Analysis of murine blood plasma for ILEI-processing capacity. Western blot analysis of purified full-length ILEI incubated with blood plasma of *Plg*-KO mice and their wild-type (wt) or heterozygous littermates. Each lane contains a plasma sample of a separate mouse. (D) *In vitro* plasmin cleavage assay of ILEI cleavage mutants. WT and cleavage-mutant (FD and DF) ILEI proteins purified via their FLAG epitope tag from lysates of overexpressing EpRas cells were incubated with purified plasmin for 24 hours and subjected to Western blot analysis. (PDF 86 KB)

Additional file 2: Figure S2.: ILEI and its processing are essential for metastasis formation of murine 4T1 mammary cancer cells. (A) ILEI Western blot analysis of whole-cell lysates and CM of parental 4T1 cells, control (shCont) and ILEI KD (shILEI) 4T1 cells and ILEI KD 4T1 cells reconstituted with wild-type (wt^rescue^), cleavage-mutant (FD^rescue^) and Δ-propeptide (ΔN-RS^rescue^) ILEI constructs. The last lane of the CM blot was inserted from a separate part of the same gel. (B) Tumor masses ± SEM 30 days after injection of 4T1 cells and derivatives into the mammary gland fat pads of female nude mice (*n* = 6 to 10 per group). (C) Histological analysis of lung metastases of mice that received injections into the fat pad. Scale bar, 2 mm. (D) The percentage of lung metastatic area ± SEM for each group. (PDF 574 KB)

Additional file 3: Figure S3.: Induction of ILEI secretion by plasmin, TGFβ and uPA and efficient uPAR KD by stable RNAi in EpRas cells. (A) Western blot analysis of ILEI in whole-cell lysates and CM of EpRas cells not treated or treated with purified plasmin of the indicated concentrations for 24 hours after serum withdrawal. (B) Western blot analysis of ILEI in whole-cell lysates and CM of EpRas cells without treatment or following TGFβ-1 (10 ng/ml), plasmin (10 mU/ml) or a combined treatment for 16 hours after serum withdrawal or reduction to 4%. (C) Western blot analysis of ILEI in whole-cell lysates and CM of EpRas cells harvested 24 hours after serum withdrawal and incubated with recombinant TGFβ-1 (10 ng/ml) or purified plasmin (10 mU/ml) for the indicated periods of time before harvest. (D) Western blot analysis of ILEI in whole-cell lysates and CM of EpRas cells not treated or treated with purified active uPA of the indicated concentrations for 24 hours after serum withdrawal. (E) Relative uPAR mRNA expression of EpRas cells stably expressing nontargeting (shCont) or uPAR-targeting shRNAs (sh_uPAR 1 to 5) determined by quantitative RT-PCR and normalized to GAPDH mRNA levels. Error bars show standard deviations of triplicates. (PDF 192 KB)

Additional file 4: Figure S4.: Expression of components of the Plg-uPAR system correlates with the extent and plasmin-dependent inducibility of ILEI secretion in human breast cancer cell lines. (A) ILEI expression and secretion levels shown by Western blot analysis of whole-cell lysates and CM of MCF7, T47D, MDA-MB-468, MDA-MB-231 and CAMA1 cells. Last two lanes of each blot were inserted from a separate part of the same gel. (B) Relative uPAR, uPA, tPA, PAI-1 and PAI-2 mRNA expression levels of MCF7, T47D, MDA-MB-468, MDA-MB-231 and CAMA1 cells determined by quantitative RT-PCR and normalized to GAPDH mRNA levels. Error bars indicate mean ± SEM of three independent experiments. (C) Western blot analysis of ILEI expression and secretion levels of whole-cell lysates and CM of MCF7 and MDA-MB-231 cells cultured in 4% FCS containing medium for 24 hours in the absence or presence of plasmin (10 mU/ml). (PDF 104 KB)

Additional file 5: Figure S5.: ILEI primarily colocalizes with Golgi and trans-Golgi network secretory organelles, but not with endosomal or degradatory compartments. Immunofluorescence analysis of subcellular ILEI localization in EpC40-wt (left panel), EpC40-ΔN-RS (middle panel) and EpC40-FD (right panel) cells. ILEI (green) was detected by using an ILEI-specific antibody. Markers of different cellular compartments (red) are visualized by specific antibodies (giantin, TGN38, EEA1 and Lamp1) or by autofluorescence following transient delivery of a fluorochrome-coupled protein (transferrin) or transient expression of an mCherry fusion protein (Rab8a). Genomic DNA (blue) is counterstained with DAPI. Scale bar, 10 μm. (PDF 275 KB)

Additional file 6: Figure S6.: Analysis of the prognostic power of ILEI, uPAR and a combined marker analysis in human breast cancer subtypes. (A) Kaplan-Meier plots depicting metastasis-free survival of patients evaluated for the four major breast cancer subtypes. (B) Kaplan-Meier plots depicting metastasis-free survival of patients of each breast cancer subtype evaluated for ILEI localization (left panels), tumor cell–associated uPAR expression (middle panels) and a combined analysis of the two markers (right panels). (PDF 116 KB)

Additional file 7: Table S1.: Correlation of ILEI and uPAR IHC status with established clinical and histopathological parameters of human breast cancer. (PDF 61 KB)

Below are the links to the authors’ original submitted files for images.

Authors’ original file for figure 1

Authors’ original file for figure 2

Authors’ original file for figure 3

Authors’ original file for figure 4

Authors’ original file for figure 5

Authors’ original file for figure 6

Authors’ original file for figure 7

## Abbreviations

CM: Conditioned medium
ECM: Extracellular matrix
EMT: Epithelial-to-mesenchymal transition
ER: Estrogen receptor
HER2: Human epidermal growth factor receptor 2
ILEI: Interleukin-like epithelial-to-mesenchymal transition inducer
KD: Knockdown
NE: Neutrophil elastase
PAI: Plasminogen activator inhibitor
Plg: Plasminogen
PR: Progesterone receptor
RNAi: RNA interference
shRNA: Short-hairpin RNA
TGN: Trans-Golgi network
tPA: Tissue plasminogen activator
uPA: Urokinase plasminogen activator
uPAR: Urokinase plasminogen activator receptor
