# Supplementary material for: Interleukin-like epithelial-to-mesenchymal transition inducer activity is controlled by proteolytic processing and plasminogen–urokinase plasminogen activator receptor system–regulated secretion during breast cancer progression
Source: Breast Cancer Res. 2014 Sep 9;16:433. doi: 10.1186/s13058-014-0433-7 (PMC4303039; doi:10.1186/s13058-014-0433-7)
Supplement: Supplementary file 1 — Additional file 1: Figure S1.: Loss of Plg partially impairs ILEI-processing capacity of serum and plasma, and mutation of the proteolytic cleavage site in ILEI prevents processing by plasmin. (A) Purified full-length ILEI-6xHis was incubated with medium containing 10% FCS (DMEM 10%) or CM of EpRas cells (EpRas CM) or added directly to the culture of EpRas cells (EpRas co-inc.) for 24 hours. ILEI protein was repurified via its 6xHis affinity tag and evaluated by Western blot analysis. (B) Western blot analysis of ILEI in CM of EpRas cells grown in the presence of 10% complete or Plg-depleted FCS. Loading was normalized to cell numbers. (C) Analysis of murine blood plasma for ILEI-processing capacity. Western blot analysis of purified full-length ILEI incubated with blood plasma of Plg-KO mice and their wild-type (wt) or heterozygous littermates. Each lane contains a plasma sample of a separate mouse. (D) In vitro plasmin cleavage assay of ILEI cleavage mutants. WT and cleavage-mutant (FD and DF) ILEI proteins purified via their FLAG epitope tag from lysates of overexpressing EpRas cells were incubated with purified plasmin for 24 hours and subjected to Western blot analysis. (PDF 86 KB) [file 13058_2014_433_MOESM1_ESM.pdf]

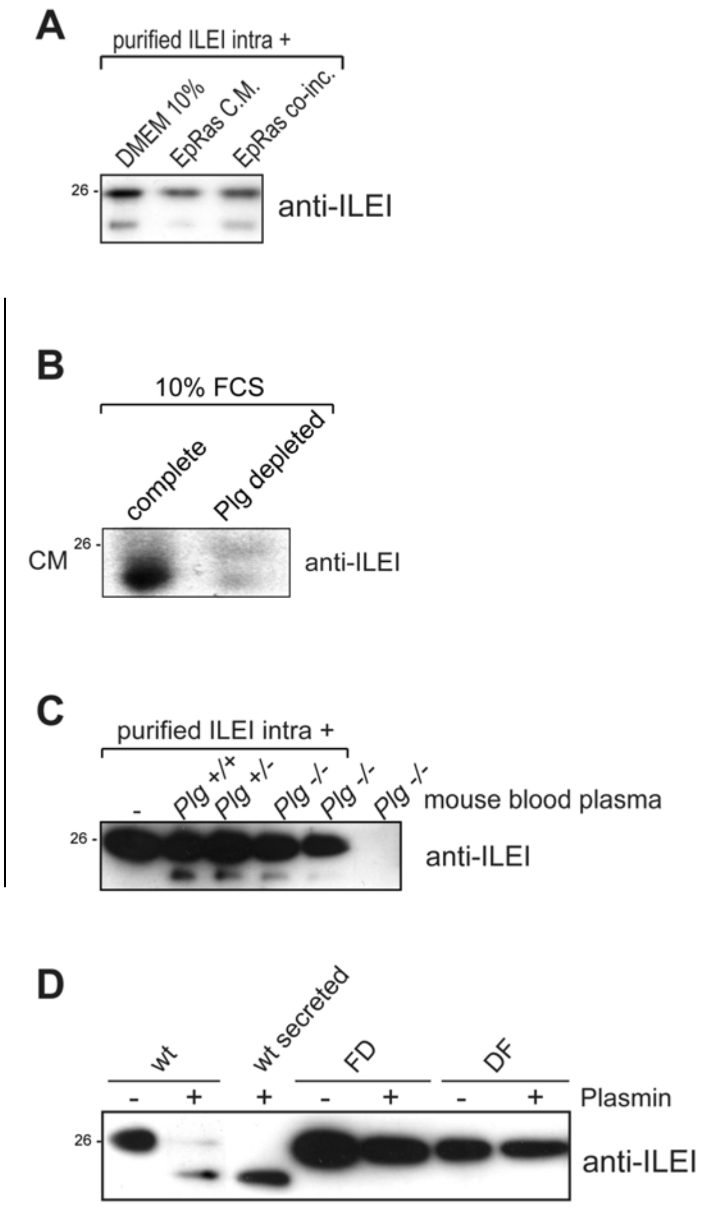

Figure S1

Figure S1. Loss of Plg partially impairs ILEI processing capacity of serum and plasma, and mutation of the proteolytic cleavage site in ILEI prevents processing by plasmin. (**A**) Purified full-lengh ILEI-6xHis was incubated with medium containing 10% FCS (DMEM 10%) or CM of EpRas cells (EpRas CM) or added directly to the culture of EpRas cells (EpRas co-inc.) for 24 hours. ILEI protein was re-purified via its 6xHis affinity tag and analyzed on Western blot. (**B**) Western blot analysis of ILEI in CM of EpRas cells grown in the presence of 10% complete or Plg-depleted FCS. Loading was normalized to cell numbers. (**C**) Analysis of murine blood plasma for ILEI processing capacity. Western blot analysis of purified full length ILEI incubated with blood plasma of *Plg* KO mice and their wild type or heterozygous littermates. Each lane contains plasma sample of a separate mouse. (**D**) In vitro plasmin cleavage assay of ILEI cleavage mutants. Wild type (wt) and cleavage mutant (FD and DF) ILEI proteins purified via their FLAG epitope tag from lysates of overexpressing EpRas cells were incubated with purified plasmin for 24 hours and analyzed on Western blot.
