# Supplementary material for: Interleukin-like epithelial-to-mesenchymal transition inducer activity is controlled by proteolytic processing and plasminogen–urokinase plasminogen activator receptor system–regulated secretion during breast cancer progression
Source: Breast Cancer Res. 2014 Sep 9;16:433. doi: 10.1186/s13058-014-0433-7 (PMC4303039; doi:10.1186/s13058-014-0433-7)
Supplement: Supplementary file 2 — Additional file 2: Figure S2.: ILEI and its processing are essential for metastasis formation of murine 4T1 mammary cancer cells. (A) ILEI Western blot analysis of whole-cell lysates and CM of parental 4T1 cells, control (shCont) and ILEI KD (shILEI) 4T1 cells and ILEI KD 4T1 cells reconstituted with wild-type (wtrescue), cleavage-mutant (FDrescue) and Δ-propeptide (ΔN-RSrescue) ILEI constructs. The last lane of the CM blot was inserted from a separate part of the same gel. (B) Tumor masses ± SEM 30 days after injection of 4T1 cells and derivatives into the mammary gland fat pads of female nude mice (n = 6 to 10 per group). (C) Histological analysis of lung metastases of mice that received injections into the fat pad. Scale bar, 2 mm. (D) The percentage of lung metastatic area ± SEM for each group. (PDF 574 KB) [file 13058_2014_433_MOESM2_ESM.pdf]

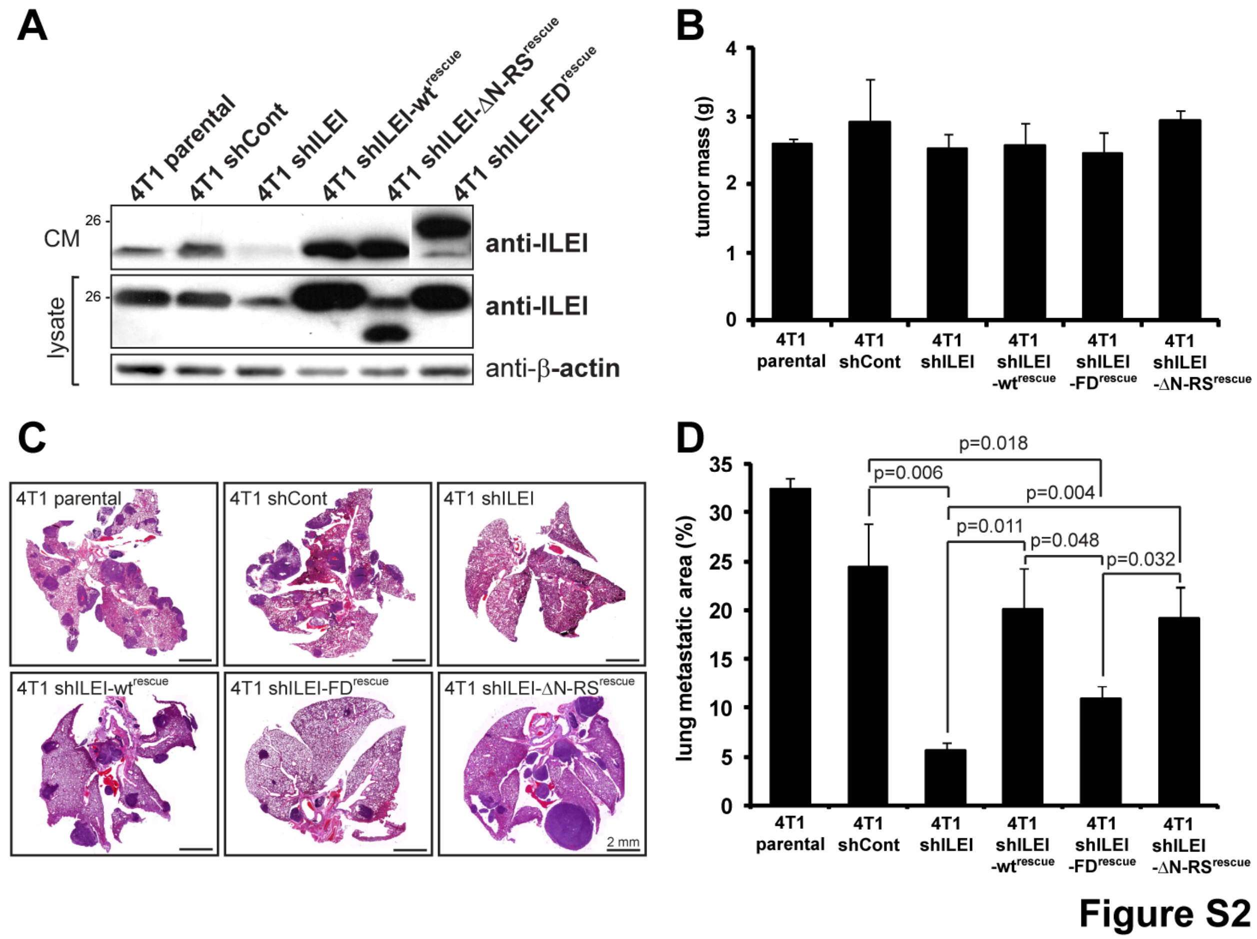

Figure S2. ILEI and its processing are essential for metastasis formation of murine 4T1 mammary cancer cells. (**A**) ILEI Western blot analysis of whole cell lysates and CM of parental 4T1 cells, control (shCont) and ILEI KD (shILEI) 4T1 cells and ILEI KD 4T1 cells reconstituted with wild type (wt^{rescue}), cleavage-mutant (FD^{rescue}) and Δpropeptide ($ΔN\text{-}RS^{rescue}$) ILEI constructs. The last lane of the CM blot was inserted from a separate part of the same gel. (**B**) Tumor masses ± SEM 30 days after injection of 4T1 cells and derivatives into the mammary gland fat pads of female nude mice (*n*=6-10 per group). (**C**) Histological analysis of lung metastases of fat pad injected mice. Scale bar, 2mm. (**D**) The percentage of lung metastatic area ± SEM for each group.
