# Supplementary material for: Interleukin-like epithelial-to-mesenchymal transition inducer activity is controlled by proteolytic processing and plasminogen–urokinase plasminogen activator receptor system–regulated secretion during breast cancer progression
Source: Breast Cancer Res. 2014 Sep 9;16:433. doi: 10.1186/s13058-014-0433-7 (PMC4303039; doi:10.1186/s13058-014-0433-7)
Supplement: Supplementary file 3 — Additional file 3: Figure S3.: Induction of ILEI secretion by plasmin, TGFβ and uPA and efficient uPAR KD by stable RNAi in EpRas cells. (A) Western blot analysis of ILEI in whole-cell lysates and CM of EpRas cells not treated or treated with purified plasmin of the indicated concentrations for 24 hours after serum withdrawal. (B) Western blot analysis of ILEI in whole-cell lysates and CM of EpRas cells without treatment or following TGFβ-1 (10 ng/ml), plasmin (10 mU/ml) or a combined treatment for 16 hours after serum withdrawal or reduction to 4%. (C) Western blot analysis of ILEI in whole-cell lysates and CM of EpRas cells harvested 24 hours after serum withdrawal and incubated with recombinant TGFβ-1 (10 ng/ml) or purified plasmin (10 mU/ml) for the indicated periods of time before harvest. (D) Western blot analysis of ILEI in whole-cell lysates and CM of EpRas cells not treated or treated with purified active uPA of the indicated concentrations for 24 hours after serum withdrawal. (E) Relative uPAR mRNA expression of EpRas cells stably expressing nontargeting (shCont) or uPAR-targeting shRNAs (sh_uPAR 1 to 5) determined by quantitative RT-PCR and normalized to GAPDH mRNA levels. Error bars show standard deviations of triplicates. (PDF 192 KB) [file 13058_2014_433_MOESM3_ESM.pdf]

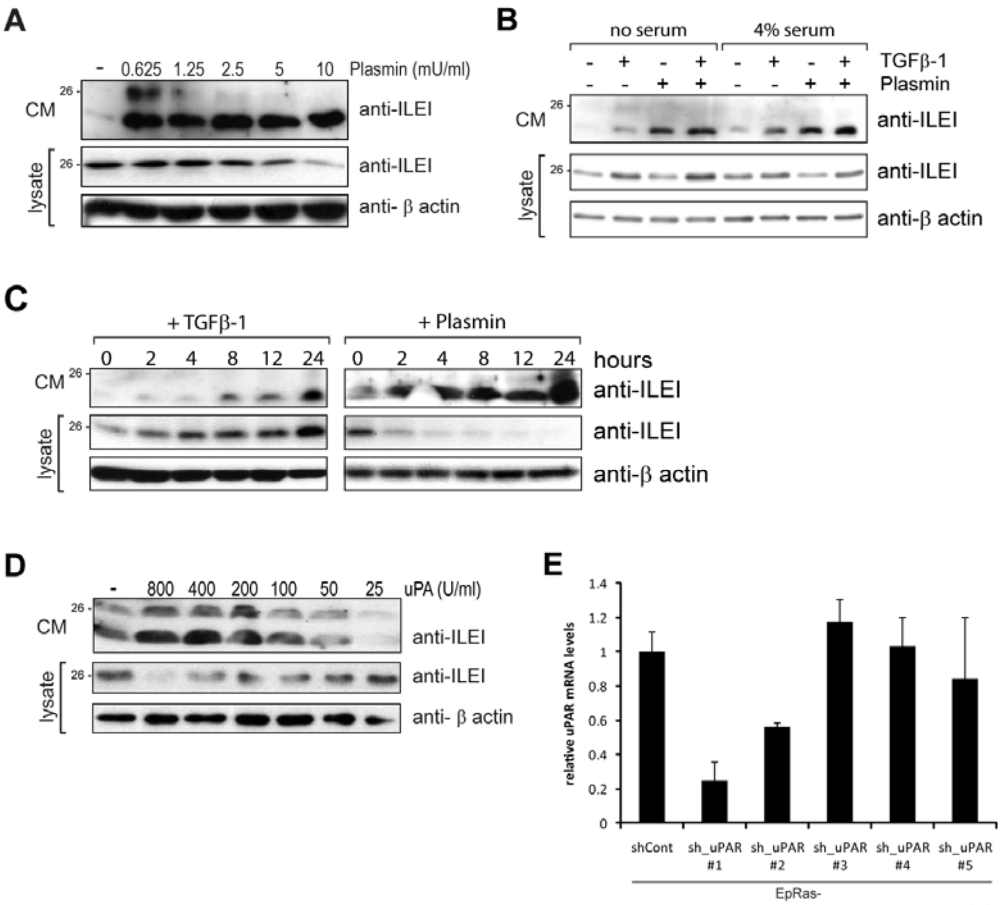

Figure S3

Figure S3. Induction of ILEI secretion by plasmin, TGFβ and uPA and efficient uPAR KD by stable RNAi in EpRas cells. (A) Western blot analysis of ILEI in whole cell lysates and CM of EpRas cells non-treated or treated with purified plasmin of the indicated concentrations for 24 hours after serum withdrawal. (B) Western blot analysis of ILEI in whole cell lysates and CM of EpRas cells without treatment or following TGFβ-1 (10 ng/ml), plasmin (10 mU/ml) or a combined treatment for 16 hours after serum withdrawal or reduction to 4%. (C) Western blot analysis of ILEI in whole cell lysates and CM of EpRas cells harvested 24 hours after serum withdrawal and incubated with recombinant TGFβ-1 (10 ng/ml) or purified plasmin (10 mU/ml) for the indicated periods of time before harvest. (D) Western blot analysis of ILEI in whole cell lysates and CM of EpRas cells non-treated or treated with purified active uPA of the indicated concentrations for 24 hours after serum withdrawal. (E) Relative uPAR mRNA expression of EpRas cells stably expressing non-targeting (shCont) or uPAR-targeting shRNAs (sh_uPAR #1-#5) determined by quantitative real-time PCR and normalized to GAPDH mRNA levels. Error bars show standard deviation of triplicates.
