# Supplementary material for: Interleukin-like epithelial-to-mesenchymal transition inducer activity is controlled by proteolytic processing and plasminogen–urokinase plasminogen activator receptor system–regulated secretion during breast cancer progression
Source: Breast Cancer Res. 2014 Sep 9;16:433. doi: 10.1186/s13058-014-0433-7 (PMC4303039; doi:10.1186/s13058-014-0433-7)
Supplement: Supplementary file 4 — Additional file 4: Figure S4.: Expression of components of the Plg-uPAR system correlates with the extent and plasmin-dependent inducibility of ILEI secretion in human breast cancer cell lines. (A) ILEI expression and secretion levels shown by Western blot analysis of whole-cell lysates and CM of MCF7, T47D, MDA-MB-468, MDA-MB-231 and CAMA1 cells. Last two lanes of each blot were inserted from a separate part of the same gel. (B) Relative uPAR, uPA, tPA, PAI-1 and PAI-2 mRNA expression levels of MCF7, T47D, MDA-MB-468, MDA-MB-231 and CAMA1 cells determined by quantitative RT-PCR and normalized to GAPDH mRNA levels. Error bars indicate mean ± SEM of three independent experiments. (C) Western blot analysis of ILEI expression and secretion levels of whole-cell lysates and CM of MCF7 and MDA-MB-231 cells cultured in 4% FCS containing medium for 24 hours in the absence or presence of plasmin (10 mU/ml). (PDF 104 KB) [file 13058_2014_433_MOESM4_ESM.pdf]

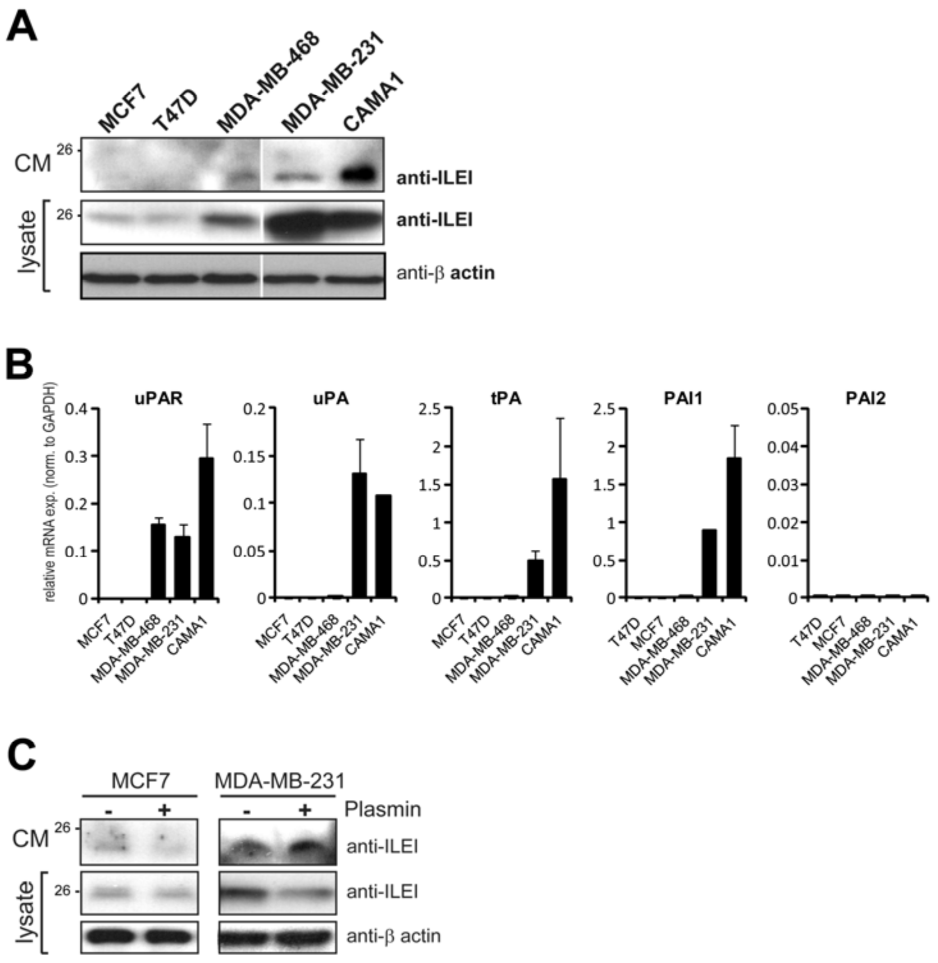

Figure S4

Figure S4. **Expression of components of the Plg-uPAR system correlates with the extent and plasmin-dependent inducibility of ILEI secretion in human breast cancer cell lines** (**A**) ILEI expression and secretion levels shown by Western analysis of whole cell lysates and CM of MCF7, T47D, MDA-MB-468, MDA-MB- 231 and CAMA1 cells. Last two lanes of each blot are inserted from a separate part of the same gel (**B**) Relative uPAR, uPA, tPA, PAI1 and PAI2 mRNA expression levels of MCF7, T47D, MDA-MB-468, MDA-MB-231 and CAMA1 cells determined by quantitative real-time PCR and normalized to GAPDH mRNA levels. Error bars indicate mean ± SEM of three independent experiments. (**C**) Western blot analysis of ILEI expression and secretion levels of whole cell lysates and CM of MCF7 and MDA-MB-231 cells cultured in 4% FCS containing medium for 24 hours in the absence or presence of plasmin (10 mU/ml).
