# Supplementary material for: Interleukin-like epithelial-to-mesenchymal transition inducer activity is controlled by proteolytic processing and plasminogen–urokinase plasminogen activator receptor system–regulated secretion during breast cancer progression
Source: Breast Cancer Res. 2014 Sep 9;16:433. doi: 10.1186/s13058-014-0433-7 (PMC4303039; doi:10.1186/s13058-014-0433-7)
Supplement: Supplementary file 5 — Additional file 5: Figure S5.: ILEI primarily colocalizes with Golgi and trans-Golgi network secretory organelles, but not with endosomal or degradatory compartments. Immunofluorescence analysis of subcellular ILEI localization in EpC40-wt (left panel), EpC40-ΔN-RS (middle panel) and EpC40-FD (right panel) cells. ILEI (green) was detected by using an ILEI-specific antibody. Markers of different cellular compartments (red) are visualized by specific antibodies (giantin, TGN38, EEA1 and Lamp1) or by autofluorescence following transient delivery of a fluorochrome-coupled protein (transferrin) or transient expression of an mCherry fusion protein (Rab8a). Genomic DNA (blue) is counterstained with DAPI. Scale bar, 10 μm. (PDF 275 KB) [file 13058_2014_433_MOESM5_ESM.pdf]

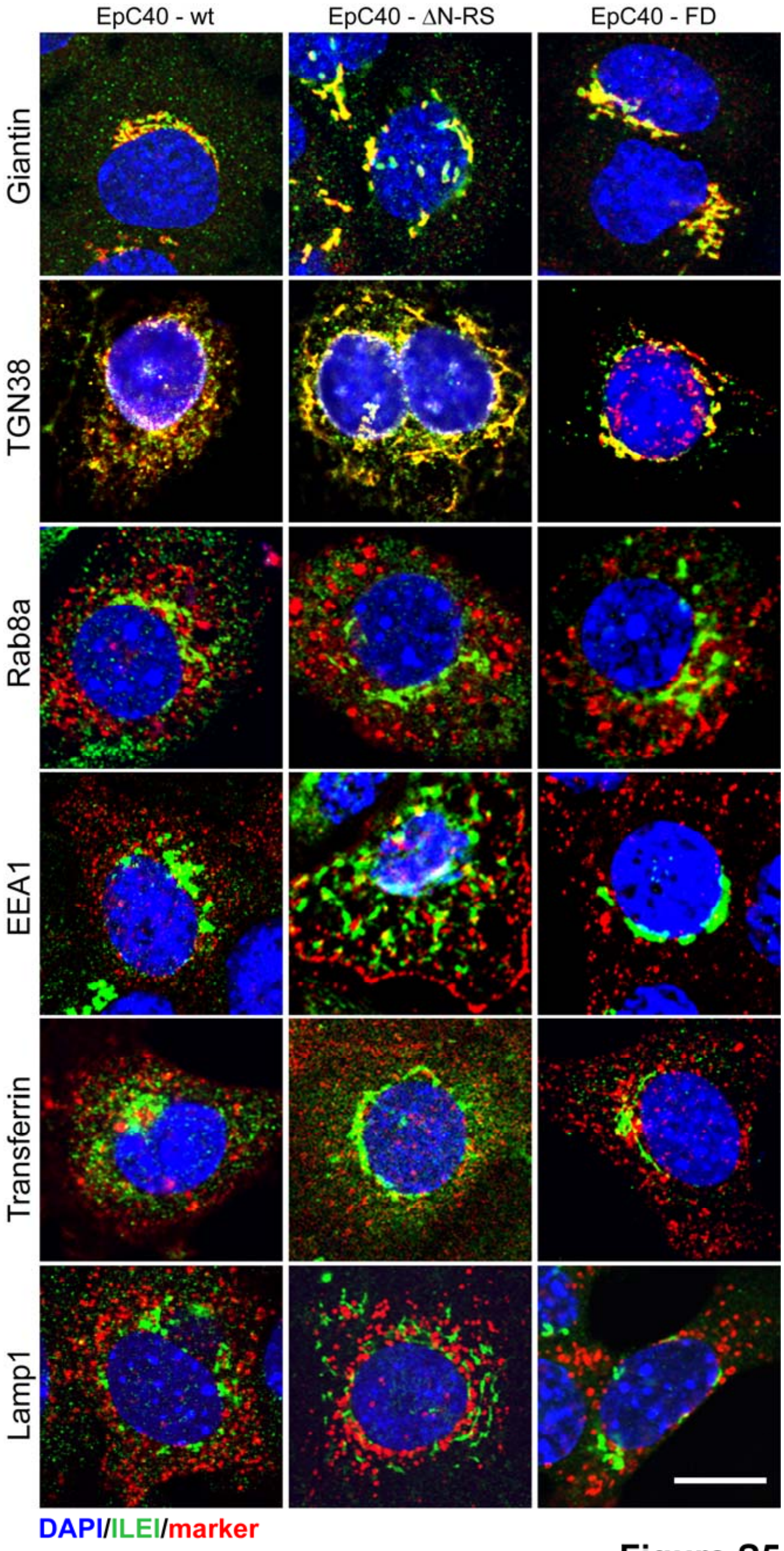

Figure S5

Figure S5. ILEI primarily co-localizes with Golgi and trans-Golgi network secretory organelles but not with endosomal or degradatory compartments. Immunofluorescent analysis of subcellular ILEI localization in EpC40-wt (left panel), EpC40-ΔN-RS (middle panel) and EpC40-FD (right panel) cells. ILEI (green) was detected via an ILEI specific antibody. Markers of different cellular compartments (red) are visualized by specific antibodies (Giantin, TGN38, EEA1 and Lamp1) or by autofluorescence following transient delivery of a fluorochrome coupled protein (Transferrin) or transient expression of an mCherry fusion protein (Rab8a). Genomic DNA (blue) is contrastained with DAPI. Scale bar, 10 µm.
