# Supplementary material for: Interleukin-like epithelial-to-mesenchymal transition inducer activity is controlled by proteolytic processing and plasminogen–urokinase plasminogen activator receptor system–regulated secretion during breast cancer progression
Source: Breast Cancer Res. 2014 Sep 9;16:433. doi: 10.1186/s13058-014-0433-7 (PMC4303039; doi:10.1186/s13058-014-0433-7)
Supplement: Supplementary file 6 — Additional file 6: Figure S6.: Analysis of the prognostic power of ILEI, uPAR and a combined marker analysis in human breast cancer subtypes. (A) Kaplan-Meier plots depicting metastasis-free survival of patients evaluated for the four major breast cancer subtypes. (B) Kaplan-Meier plots depicting metastasis-free survival of patients of each breast cancer subtype evaluated for ILEI localization (left panels), tumor cell–associated uPAR expression (middle panels) and a combined analysis of the two markers (right panels). (PDF 116 KB) [file 13058_2014_433_MOESM6_ESM.pdf]

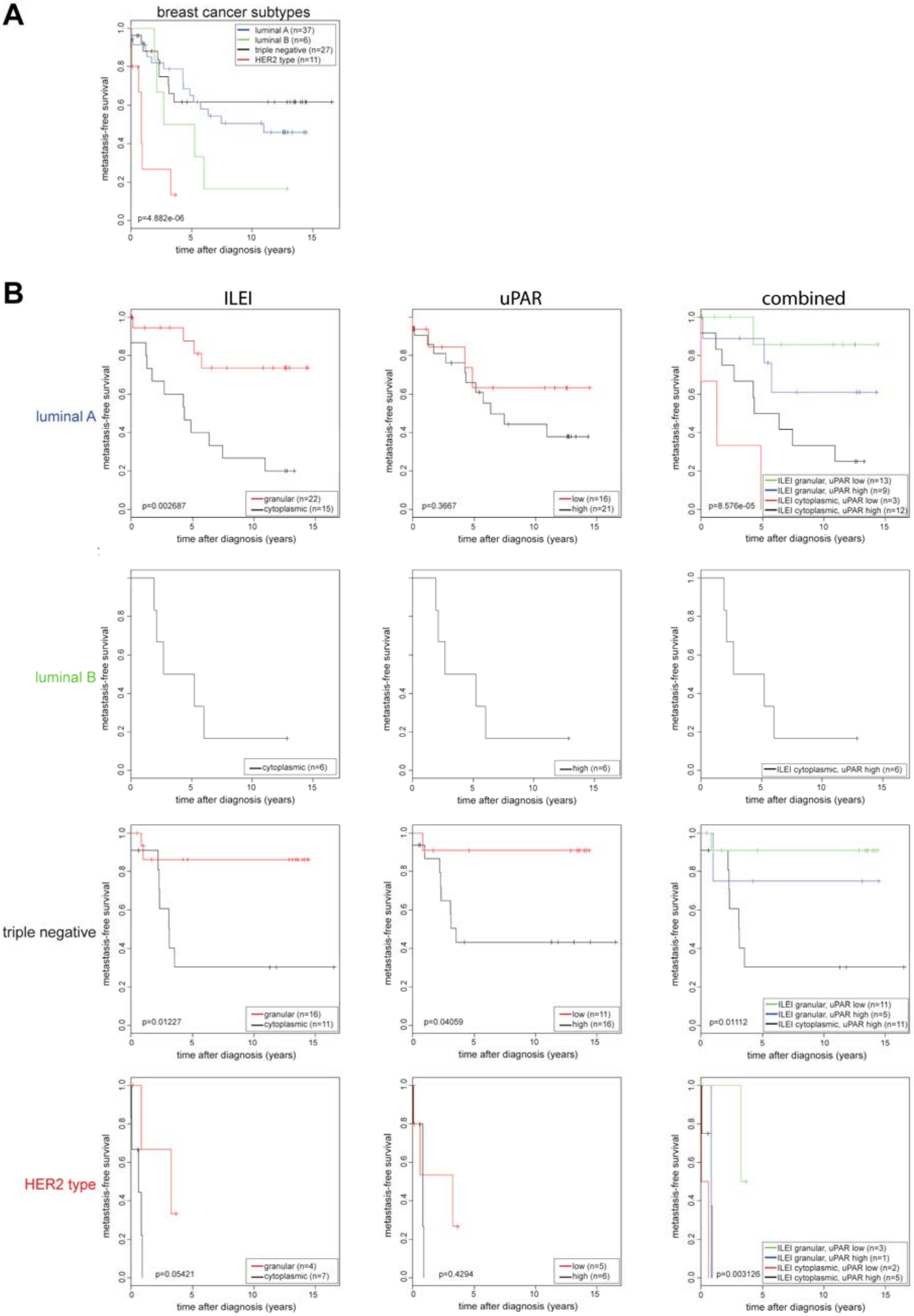
A
breast cancer subtypes
luminal A (n=37)
luminal B (n=6)
triple negative (n=27)
HER2 type (n=11)
p=4.882e-06
metastasis-free survival
time after diagnosis (years)
B
ILEI
uPAR
combined
luminal A
luminal B
triple negative
HER2 type
granular (n=22)
cytoplasmic (n=15)
p=0.002687
low (n=16)
high (n=21)
p=0.3667
ILEI granular, uPAR low (n=13)
ILEI granular, uPAR high (n=9)
ILEI cytoplasmic, uPAR low (n=3)
ILEI cytoplasmic, uPAR high (n=12)
p=8.576e-05
cytoplasmic (n=6)
high (n=6)
ILEI cytoplasmic, uPAR high (n=6)
granular (n=16)
cytoplasmic (n=11)
p=0.01227
low (n=11)
high (n=16)
p=0.04059
ILEI granular, uPAR low (n=11)
ILEI granular, uPAR high (n=5)
ILEI cytoplasmic, uPAR high (n=11)
p=0.01112
granular (n=4)
cytoplasmic (n=7)
p=0.05421
low (n=5)
high (n=6)
p=0.4294
ILEI granular, uPAR low (n=3)
ILEI granular, uPAR high (n=1)
ILEI cytoplasmic, uPAR low (n=2)
ILEI cytoplasmic, uPAR high (n=5)
p=0.003126

**Figure S6**

**Figure S6. Analysis on the prognostic power of ILEI, uPAR and a combined marker analysis in human breast cancer subtypes.** (**A**) Kaplan-Meier plots depicting metastasis-free survival of patients evaluated for the four major breast cancer subtypes, (**B**) Kaplan-Meier plots depicting metastasis-free survival of patients of each breast cancer subtype evaluated for ILEI localization (left panels), tumor-cell associated uPAR expression (middle panels) and a combined analysis of the two markers (right panels).
