# Supplementary material for: Interleukin-like epithelial-to-mesenchymal transition inducer activity is controlled by proteolytic processing and plasminogen–urokinase plasminogen activator receptor system–regulated secretion during breast cancer progression
Source: Breast Cancer Res. 2014 Sep 9;16:433. doi: 10.1186/s13058-014-0433-7 (PMC4303039; doi:10.1186/s13058-014-0433-7)
Supplement: Supplementary file 7 — Additional file 7: Table S1.: Correlation of ILEI and uPAR IHC status with established clinical and histopathological parameters of human breast cancer. (PDF 61 KB) [file 13058_2014_433_MOESM7_ESM.pdf]

Table S1. Correlation of ILEI and uPAR IHC status with established clinical and histopathological parameters of human breast cancer.

Patient parameters		ILEI / uPAR	granular low (n=28)		granular high (n=16)		cytoplasmatic low (n=6)		cytoplasmatic high (n=38)		p-value
**Age of onset**											
<50	(n=33)		9	(32.1)	5	(31.3)	3	(50.0)	16	(42.1)	0.7350
≥50	(n=55)		19	(67.9)	11	(68.8)	3	(50.0)	22	(57.9)	
**Tumor type**											
ductal	(n=65)		19	(67.9)	13	(81.3)	5	(83.3)	28	(77.8)	0.7592
lobular	(n=21)		9	(32.1)	3	(18.8)	1	(16.7)	8	(22.2)	
other, na	(n=2)		0		0		0		2		
**Tumor size**											
pT1	(n=25)		9	(32.1)	5	(31.3)	2	(33.3)	9	(25.7)	0.9052
pT2-4	(n=60)		19	(67.9)	11	(68.8)	4	(67.7)	26	(74.3)	
na	(n=3)		0		0		0		3		
**Tumor grade**											
pG1-2	(n=45)		17	(60.7)	7	(43.8)	2	(33.3)	19	(54.3)	0.5750
pG3	(n=40)		11	(39.3)	9	(56.3)	4	(66.7)	16	(45.7)	
na	(n=3)		0		0		0		3		
**Tumor stage**											
1	(n=17)		7	(26.9)	3	(21.4)	1	(16.7)	6	(18.8)	0.9066
2-4	(n=61)		19	(73.1)	11	(78.6)	5	(83.3)	26	(81.3)	
na	(n=10)		2		2		0		6		
**Lymph node status**											
pN0	(n=29)		14	(51.9)	5	(33.3)	1	(16.7)	9	(27.3)	0.1883
pN+	(n=52)		13	(48.1)	10	(66.7)	5	(83.3)	24	(72.7)	
na	(n=7)		1		1		0		5		
**ER status**											
neg	(n=50)		16	(57.1)	8	(53.3)	4	(66.7)	22	(61.1)	0.9335
pos	(n=35)		12	(42.9)	7	(46.7)	2	(33.3)	14	(38.9)	
na	(n=3)		0		1		0		2		
**PR status**											
neg	(n=54)		17	(63.0)	9	(60.0)	4	(80.0)	24	(68.6)	0.8504
pos	(n=28)		10	(37.0)	6	(40.0)	1	(20.0)	11	(31.4)	
na	(n=6)		1		1		1		3		
**HER2 status**											
neg	(n=68)		25	(89.3)	15	(93.8)	3	(50.0)	25	(69.4)	0.0271
pos	(n=18)		3	(10.7)	1	(6.3)	3	(50.0)	11	(30.6)	
na	(n=2)		0		0		0		2		
**p53 status**											
neg	(n=56)		16	(57.1)	13	(81.3)	5	(83.3)	22	(57.9)	0.2608
pos	(n=32)		12	(42.9)	3	(18.8)	1	(16.7)	16	(42.1)	
**Ki67 pos cells**											
<10%	(n=55)		16	(59.3)	11	(78.6)	4	(66.7)	24	(64.9)	0.6978
≥10%	(n=29)		11	(40.7)	3	(21.4)	2	(33.3)	13	(35.1)	
na	(n=4)		1		2		0		1		

The number of patients (n) and their relative frequencies (in %; figures in parentheses) in the indicated clinical and histophathological categories are shown. p-value, Fisher's exact test p-values; pN0, no lymph node metastases; pN+ patient has lymph node metastases; na, status not available. Note that na subjects were not included in the calculations of percentages and p-values.
