# Supplementary figures and images for: Interleukin-like epithelial-to-mesenchymal transition inducer activity is controlled by proteolytic processing and plasminogen–urokinase plasminogen activator receptor system–regulated secretion during breast cancer progression
Source: Breast Cancer Res. 2014 Sep 9;16:433. doi: 10.1186/s13058-014-0433-7 (PMC4303039; doi:10.1186/s13058-014-0433-7)

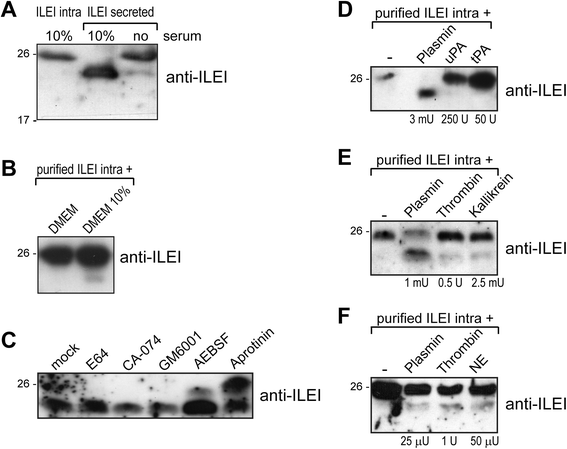

Supplement: Supplementary file 8 — Authors’ original file for figure 1 [file 13058_2014_433_MOESM8_ESM.gif]

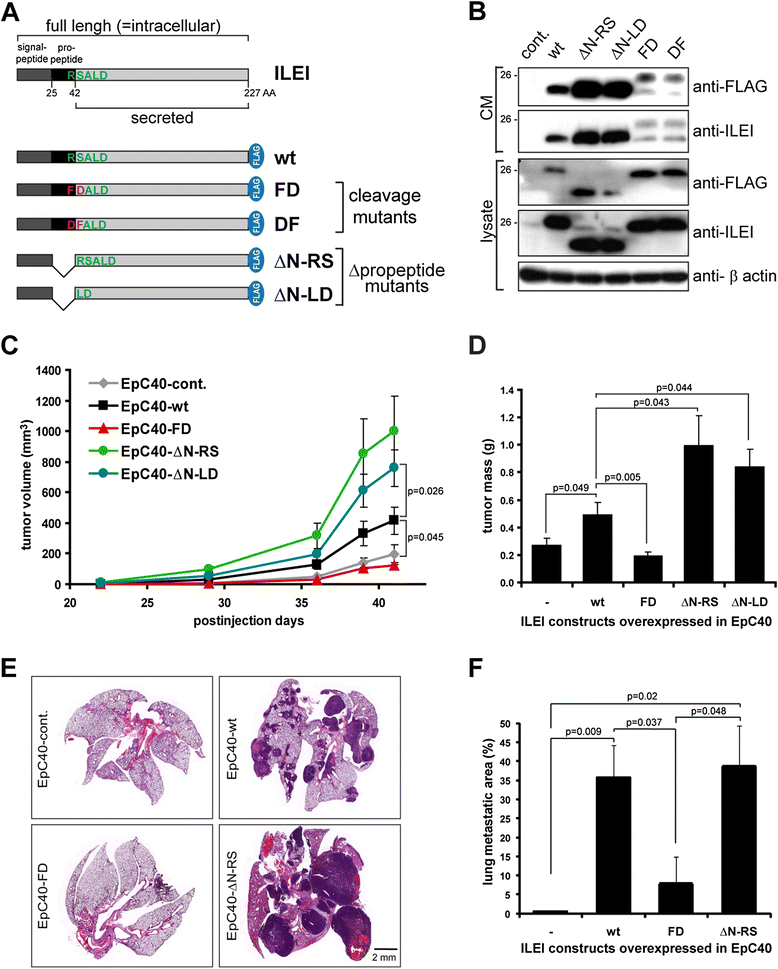

Supplement: Supplementary file 9 — Authors’ original file for figure 2 [file 13058_2014_433_MOESM9_ESM.gif]

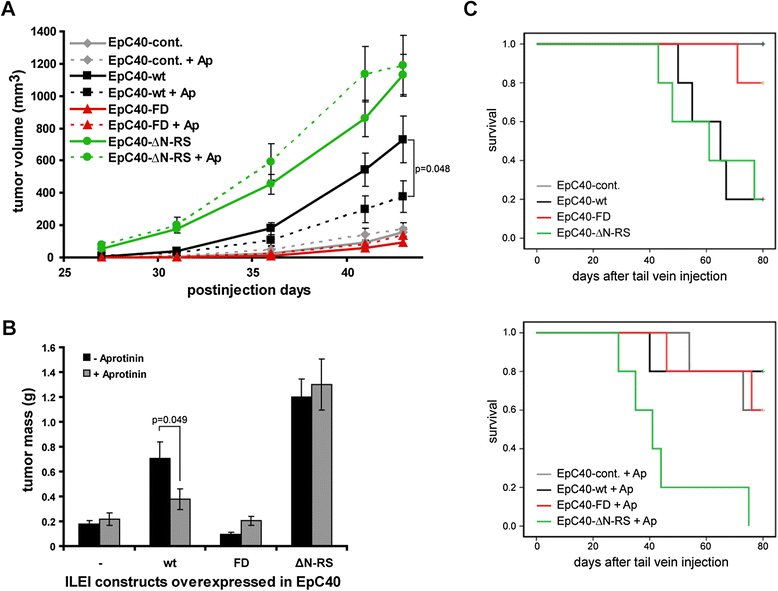

Supplement: Supplementary file 10 — Authors’ original file for figure 3 [file 13058_2014_433_MOESM10_ESM.gif]

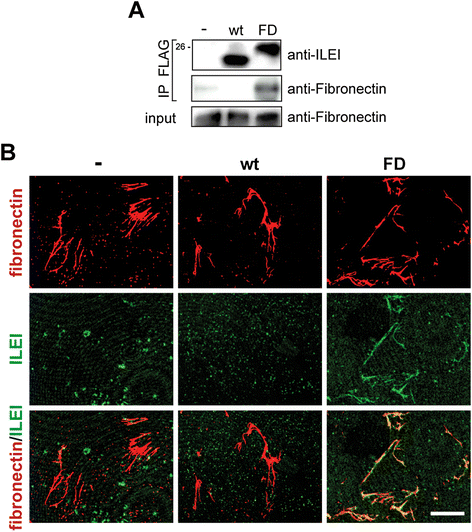

Supplement: Supplementary file 11 — Authors’ original file for figure 4 [file 13058_2014_433_MOESM11_ESM.gif]

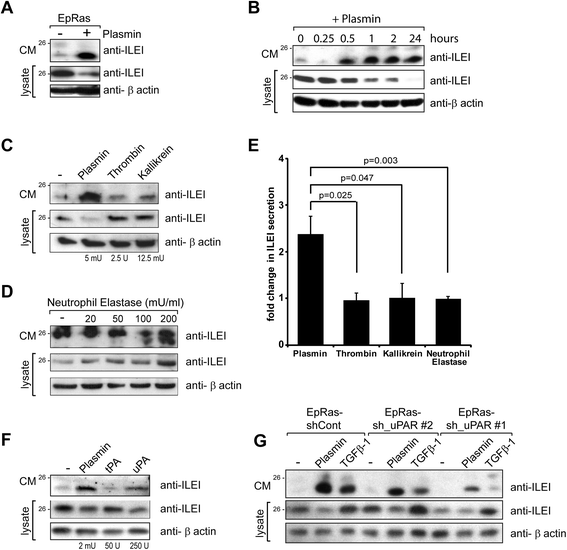

Supplement: Supplementary file 12 — Authors’ original file for figure 5 [file 13058_2014_433_MOESM12_ESM.gif]

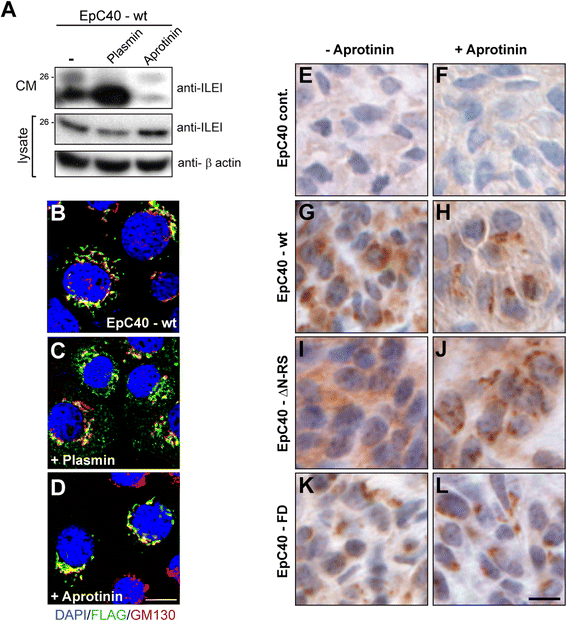

Supplement: Supplementary file 13 — Authors’ original file for figure 6 [file 13058_2014_433_MOESM13_ESM.gif]

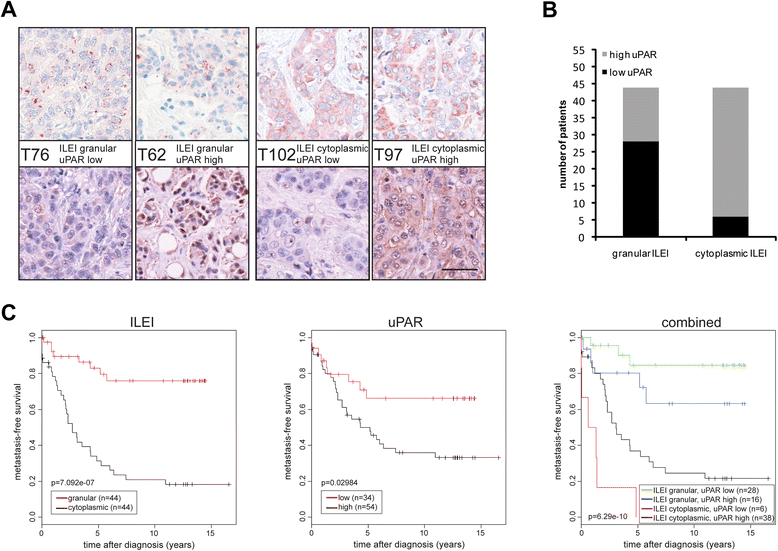

Supplement: Supplementary file 14 — Authors’ original file for figure 7 [file 13058_2014_433_MOESM14_ESM.gif]
